# From “metabolic storm” to “immune paralysis”: the dynamic evolution of macrophages and metabolism reprogramming in ARDS

**DOI:** 10.3389/fimmu.2025.1738713

**Published:** 2025-12-19

**Authors:** Jia Tang, Mi Yan, Mengchun Li, Zhangxue Hu, Kun Zhou

**Affiliations:** Department of Pediatrics, Daping Hospital, Army Medical University, Chongqing, China

**Keywords:** ARDS, macrophage polarization, metabolic reprogramming, metabolic storm, immune paralysis, glycolysis, BMMSCs, exosomes

## Abstract

Sepsis is characterized by high mortality and a complicated pathological mechanism. Macrophages play a crucial role in the initiation and progression of sepsis-associated acute respiratory distress syndrome (ARDS). Macrophage functional states and polarization phenotype have been significantly influenced by metabolic programming. This review delineates the metabolic reprogramming of macrophages from the initial ‘metabolic storm’ to subsequent ‘immune paralysis’ in sepsis-associated ARDS. It focuses on the interplay between macrophage polarization (classical activated macrophages (M1) and alternative activated macrophages (M2) phenotypes) and key metabolic pathways, including glycolysis and oxidative phosphorylation (OXPHOS). Furthermore, it explains the molecular mechanism underlying the metabolic pattern’s influence on macrophage and lung tissue damage. The final section of this review focuses on the therapeutic implications of bone marrow mesenchymal stem cells (BMMSCs) and myeloid-derived suppressor cells (MDSCs), which alter macrophage metabolic reprogramming. Based on the latest progress, this article aims to provide a comprehensive theoretical framework and cli Based on recent advances, nical guidance for immunometabolic therapy in sepsis-associated ARDS.

## 1 Introduction

Sepsis is a life-threatening syndromic entity with a critical constellation of signs and symptoms (1, 2). Sepsis results from a dysregulated host response to infection. It typically begins with an initial hyperinflammatory phase (systemic inflammatory response syndrome, SIRS), characterized by excessive pro-inflammatory cytokine release (2, 3). This can subsequently transition to a compensatory anti-inflammatory phase, leading to immunosuppression. ARDS caused by sepsis has a multi-faceted pathophysiology involving immune dysregulation, endothelial and alveolar epithelial damage, and altered metabolism, all of which compromise pulmonary function (3, 4).

Macrophages, as a central aspect of sepsis and ARDS immunopathology, are innate immune cells that activate an inflammatory response while simultaneously contributing to its suppression due to their plasticity and polarization states (5). Macrophages are classically polarized into M1 and M2 phenotypes, with proinflammatory and microbicidal properties in the former and anti-inflammatory and tissue-repair functions in the latter (5). ARDS disrupts explicitly the balance between M1 polarization support and M2 polarization, leading to metabolic acidosis and tissue damage in the alveoli through inflammation, and ultimately, to M2 polarization, which is associated with resolution and tissue recovery (6).

Metabolic reprogramming—a shift in cellular metabolic states—is intrinsically linked to macrophage polarization and function (7, 8). This includes glycolysis, OXPHOS, fatty acid oxidation (FAO), and amino acid metabolism pathways, which are associated with a proinflammatory phenotype and effector functions in distinct ways (9, 10). The Warburg effect, which links aerobic glycolysis with energy demands and proinflammatory mediator production in M1 macrophages, contrasts with OXPHOS and FAO, used to sustain anti-inflammatory functions in M2 macrophages. Metabolic reprogramming of pulmonary macrophages is a key factor that integrates bioenergetics and immune responses, influencing the progression of sepsis-induced ARDS (11, 12), which is regulated by signaling pathways and epigenetic mechanisms. Hypoxia-inducible factor (HIF-1α), a major regulator of cellular adaptation during hypoxia and metabolic stress, is involved in enhancing glycolysis and M1 macrophage polarization, which exacerbates lung inflammation (10). Other key signaling pathways include sirtuin 6 (SIRT6), which promotes anti-inflammatory macrophage polarization and autophagy in septic lungs, and ankyrin demonstrated domain 22 (ANKRD22), which enhances M1 polarization via interferon regulatory factor 3 (IRF3) in sepsis-induced ARDS (13). Non-coding RNAs and circular RNAs mediate macrophage polarization through competing endogenous RNA (ceRNA) mechanisms that regulate the inflammatory microenvironment of sepsis-ARDS (14).

“Metabolic storm” is characterized by increased glycolytic flux, reactive oxygen species (ROS) production, and M1 macrophage-driven release of proinflammatory cytokines. This is followed by “immune paralysis,” or an immunosuppressed state, in which metabolic changes, metabolic exhaustion, and M2 polarization create a barrier to pathogen clearance, thereby increasing the risk of nosocomial infections. This immune suppression, known as immunosuppression or exhaustion, underscores the need for M2 to balance M1 and M2 in defending the host and repairing tissue without causing harm.

**Figure 1:**
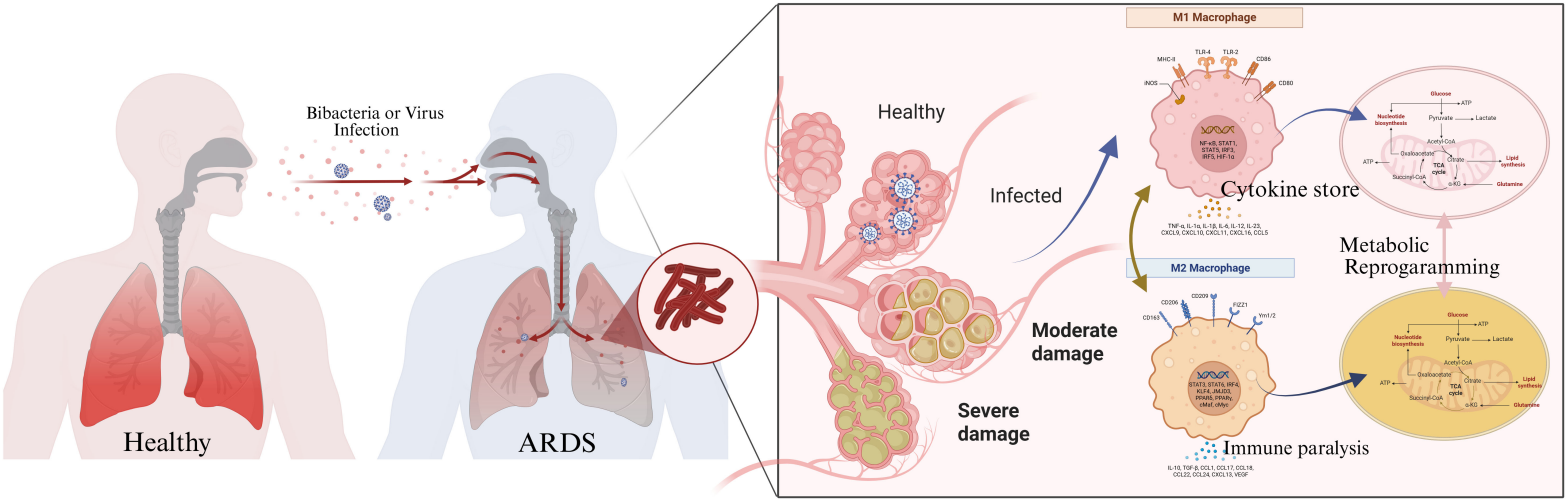
The dynamic evolution of macrophages and metabolic reprogramming in sepsis-associated ARDS. (Created with Biorender.com).

BMMSCs have been utilized to minimize glycolysis while promoting M2 polarization of alveolar macrophages (15, 16). Furthermore, it explains the molecular mechanism underlying the metabolic pattern’s influence on macrophage and lung tissue damage. The final section of this review focuses on the therapeutic implications of BMMSCs and MDSCs, which alter macrophage metabolic reprogramming. Conceptualizing the molecular and metabolic mechanisms that govern macrophage function in sepsis-ARDS as integrated network management system (INMS) provides a critical framework for elucidating pathogenesis and identifying targeted interventions. This review systematically examines the landscape alterations that occur in sepsis-ARDS, with a particular focus on macrophage metabolic reprogramming and its dynamic impact on immune modulation and clinical therapeutic implications (**Figure 1**).

## 2 Macrophage polarization in sepsis-induced ARDS

### 2.1 Dynamics of macrophage polarization in sepsis-induced ARDS

#### 2.1.1 M1 macrophages in sepsis-induced ARDS: pro-inflammatory roles and mechanisms

M1 macrophages, also known as classically activated macrophages, are crucial in the early inflammatory response because they secrete large amounts of proinflammatory cytokines, including tumor necrosis factor-alpha (TNF-α) and interleukin-1 beta (IL-1β). Macrophage polarization to the M1 phenotype is associated with increased glycolysis and the expression of proinflammatory genes, which continuously generate inflammatory signals. Apolipoprotein B mRNA editing enzyme catalytic subunit 3A (APOBEC3A), an RNA editing enzyme, encourages M1 polarization by targeting cytokine gene expression and promoting inflammatory cytokine secretion (17). This excessive proliferation of the proinflammatory response is evident in the “metabolic storm” phase of sepsis-induced ARDS, during which metabolic repolarization drives macrophage overactivation.

M1 macrophages also produce ROS and pro-oxidant enzymes, such as nicotinamide adenine dinucleotide phosphate hydrogen (NADPH) oxidase and cyclooxygenase-2, which contribute to increased oxidative stress and lipid peroxidation, they may further exacerbate tissue injury. Although some natural compounds such as carnosine can reverse these pro-oxidant and proinflammatory responses through upregulating antioxidant enzyme expression and reducing IL-1β levels, opportunities exist for novel therapeutic strategies to suppress M1 macrophage-induced inflammation Also, the inflammatory behavior of M1 macrophages is not fixed; indeed, interleukin (IL)-21, acts to impair M1 inflammatory capabilities, meaning that the cytokine environment plays a crucial role in determining M1 macrophage performance (18, 19). In pathological conditions, such as sepsis and ARDS, M1 macrophages contribute to alveolar barrier disruption by secreting TNF-α and IL-1β, which increase vascular permeability and promote leukocyte recruitment. This is exemplified by studies showing that vascular endothelial growth factor-A (VEGFA), which is upregulated in ARDS, drives M1 polarization and the concomitant release of proinflammatory cytokines, thereby exacerbating lung injury (20).

M1 macrophages produce high levels of hydrogen peroxide and other ROS, which sustain oxidative stress and tissue damage in inflamed lungs by collaborating with neutrophil-derived myeloperoxidase (21). The metabolic reprogramming that facilitates the proinflammatory phenotype of M1 macrophages is the adaptation of glycolysis. This is evidenced in the regulation of glucose transporter one and inflammatory mediators associated with M1 polarization (22). Inflamed tissues are hypoxic, which further increases M1 polarization through c-Jun N-terminal kinase (JNK)/p65 signaling pathways (23). The pathological role of M1 macrophages is observed in chronic inflammatory diseases and autoimmune conditions, all of which share tissue damage and fibrosis. Sulforaphene is a therapeutic agent that targets the NOD-like receptor (NLR) inflammasome, specifically the NLRP3 inflammasome, thereby inhibiting M1 polarization and inflammation (24).

#### 2.1.2 M2 macrophages in sepsis-induced ARDS: anti-inflammatory and reparative functions

M2 Macrophages are of paramount importance due to their role in inflammation suppression and tissue repair. In this regard, this type is instrumental in sepsis-induced ARDS, in which a person’s immune response transitions from hyperactivity to immune suppression. The type secretes anti-inflammatory cytokines, such as interleukin-10 (IL-10) and transforming growth factor beta, which are instrumental in reducing inflammation. M2 macrophage is not homogeneous. Instead, it comprises several subtypes with distinct functions, almost all of which are linked to immunosuppression and repair. M2a and M2c subtypes primarily mediate the anti-inflammatory response and tissue regeneration. M2b reservoirs possess both proinflammatory and immunoregulatory characteristics. The switch to the M2 phenotype is supported by various metabolic repurposing and signaling pathways, including FAO and respiration, that maintain their repair functions (25–28). The adverse effects of abundant M2 polarization *in vivo* are worsened during the sepsis immunosuppressive phase.

Research indicates that macrophage metabolism in sepsis displays distinct mixed characteristics, encompassing both the enhanced glycolysis typical of M1 macrophages and the OXPHOS metabolic components characteristic of M2 macrophages. This metabolic cross-regulation not only reflects macrophages’ adaptive adjustments to energy metabolism within the complex microenvironment of sepsis but also underscores their functional diversity and heterogeneity (29). During the immune response to sepsis, macrophages demonstrate a high degree of plasticity and heterogeneity in their metabolic state and function. The synergistic activation of dual signaling molecules plays a pivotal role in modulating inflammatory metabolic pathways. High mobility group box 1 protein (HMGB1) and cold-inducible RNA-binding protein (CIRP), as unique signaling molecules in sepsis, jointly activate the toll-like receptor 4 (TLR4)/nuclear factor kappa-light-chain-enhancer of activated B cells (NF-κB) and HIF-1a signaling pathways within macrophages, thereby enhancing both glycolytic metabolism and the pro-inflammatory response. Furthermore, these two signaling molecules also activate HIF-1α, a transcription factor that is central to the metabolic reprogramming of macrophages. It promotes the expression of key glycolytic enzymes, boosts the activity of the glycolytic pathway, and provides a metabolic foundation for rapid energy provision and the synthesis of inflammatory mediators in macrophages (30).

#### 2.1.3 M1/M2 polarization imbalance and ARDS progression

**Figure 2:**
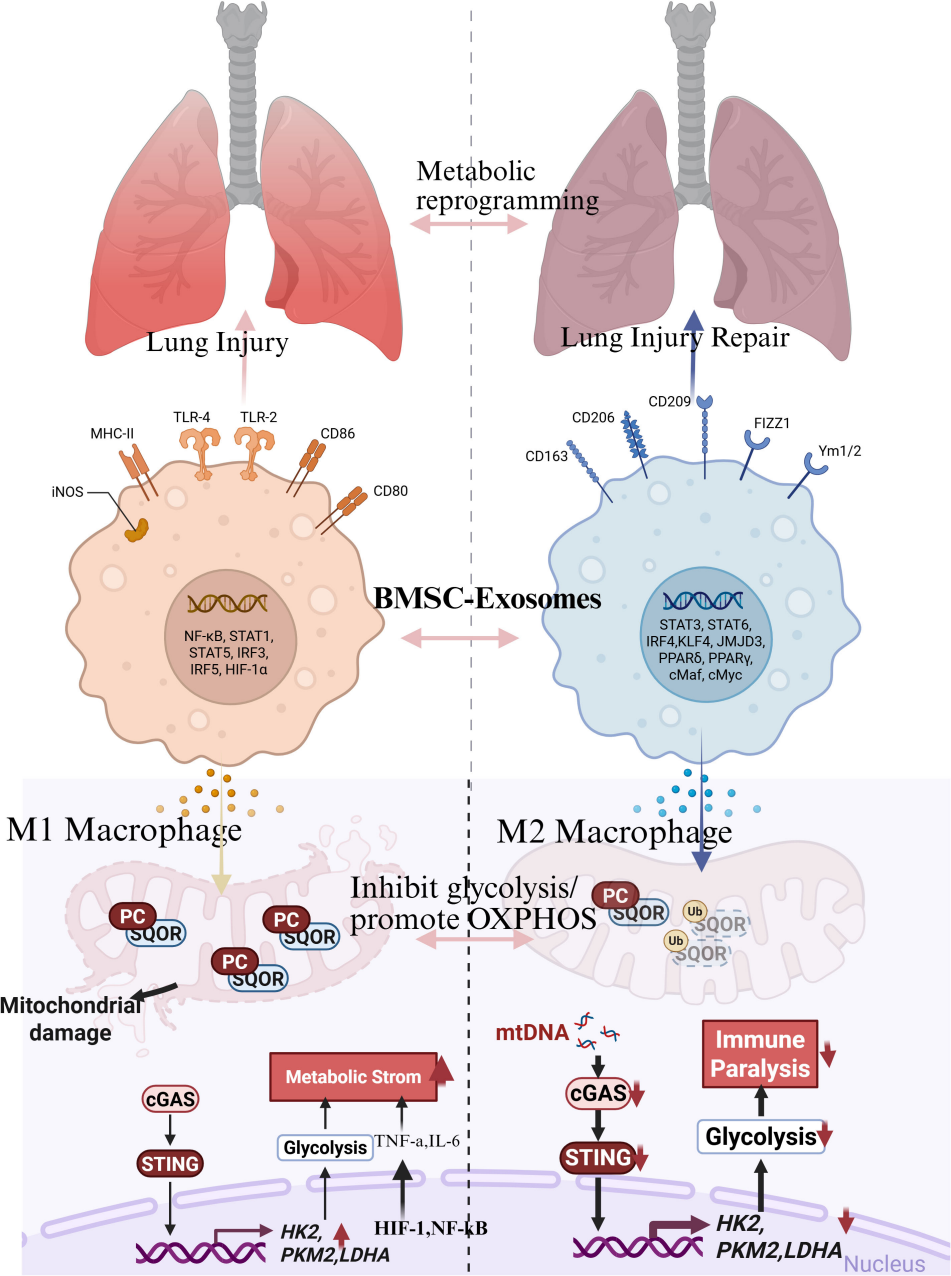
From “metabolic storm” to “immune paralysis”: the dynamic evolution of macrophages and metabolic reprogramming in sepsis-associated ARDS. (Created with Biorender.com).

The M1 and M2 polarization types in macrophages, as previously described, are key factors in the development and progression of ARDS. The balance between these two types mainly governs the inflammatory phase, tissue repair, and clearance phase. In ARDS patients, lung macrophages show a higher M1/M2 ratio due to the overexpression of secreted phosphoprotein 1 (SPP1). This gene increases the ratio by inhibiting HIF-1α, thereby exacerbating lung injury and fibrosis in ARDS models (31, 32). Furthermore, DAMPs can exacerbate inflammation by disrupting the member of the Ras oncogene family (Rab26) maturation following erythropoietin receptor (EPOR) activation and suppressing M2 polarization, thereby prolonging the inflammatory response (33) (**Figure 2**).

Otherwise, therapeutic interventions targeting macrophage polarization have illustrated promise. As previously discussed, mesenchymal stem cells (MSCs) and their exosomes can initiate M2 polarization by inhibiting glycolysis, thereby improving outcomes in ARDS (34). Additionally, pharmacological agents such as rhein and luteolin have been demonstrated to promote M2 polarization (35, 36). Similarly, CD274 has been reported to act as a negative regulator of M1 polarization while promoting an M2 phenotype, suggesting a potential new diagnostic or therapeutic approach (37). Furthermore, due to its dynamic plasticity, metabolic reprogramming is another key biological feature, with FAO and glycolysis influencing the macrophages’ phenotype and function, especially in the context of ARDS (38–41) reported that the neuropeptide calcitonin gene-related peptide partially corrected the imbalance of M1/M2 in bronchoalveolar lavage fluid from ARDS patients. Sepsis is characterized by a high degree of heterogeneity in its pathological features, particularly in terms of the metabolic reprogramming and functional manifestations of macrophages.

### 2.2 Molecular mechanisms of macrophage metabolic reprogramming

#### 2.2.1 Enhanced glycolysis drives M1 polarization

Proinflammatory M1 macrophages heavily rely on enhanced glycolysis to rapidly generate energy for synthesizing and secreting inflammatory mediators. This metabolic reprogramming supports their effector functions during acute inflammatory responses. When activated by stimuli such as lipopolysaccharide (LPS) and interferon-gamma (IFN-γ), macrophages shift from OXPHOS to aerobic glycolysis, known as the Warburg effect, to meet the increased bioenergetic and biosynthetic demands of inflammation. This glycolytic shift enables faster adenosine triphosphate (ATP) production, albeit less efficiently, allowing rapid responses to pathogens or tissue injury (42). Key glycolytic enzymes, including hexokinase 2 (HK2), pyruvate kinase M2 (PKM2), phosphofructokinase-2/fructose-2,6-bisphosphatase 3 (PFKFB3), and lactate dehydrogenase A (LDHA), are upregulated during M1 polarization, enhancing glucose uptake and lactate production (43, 44). Following these metabolic reprogramming events, the transcription factor HIF-1α plays a vital role in regulating cellular processes. HIF-1α activates glycolytic gene expression. Under inflammatory conditions, HIF-1α is stabilized in a tumor, even under normoxia, through signaling pathways such as the Janus kinase 2 (JAK2)/signal transducer and activator of transcription 1 (STAT1) axis, activated by IFN-γ, or the nuclear factor kappa-light-chain-enhancer of activated B cells (NF-κB) and mTORC1 pathways (45). The JAK2/STAT1 signaling pathway induced by IFN-γ promotes PFKFB3 expression, thereby boosting Glycolysis and M1 polarization in vascular inflammation. Similarly, the JNK/Cyclooxygenase-2 (COX-2)/HIF-1α axis enhances glycolysis in the M1 polarization of human immunodeficiency virus (HIV)-1-infected macrophages, linking metabolic reprogramming to chronic inflammation (46). This metabolic pathway not only supplies energy but also produces intermediates that act as signaling molecules to upregulate inflammatory genes. Nitric oxide (NO) produced by inducible nitric oxide synthase (iNOS) stabilizes HIF-1α, promoting glycolysis and inflammatory cytokine production (47). Additionally, pyruvate kinase M2 is a key regulator of glycolysis and inflammatory gene expression in M1 macrophages, switching between its tetrameric enzymatic form and dimeric transcriptional coactivator form (48). Several endogenous and exogenous factors influence this glycolytic reprogramming. RNA-binding motif protein 4 (RBM4) inhibits M1 polarization by suppressing STAT1-mediated glycolysis (49). Natural compounds such as Cassiaside C and berberine decrease M1 polarization by inhibiting glycolysis through HIF-1α and mTORC1 signaling, respectively, and by activating AMP-activated protein kinase (AMPK) (50). Factors like C-X-C motif chemokine receptor 1 (CXCR4) support M1 polarization by promoting glycolysis via the phosphatidylinositol-3 kinase (PI3K)/protein kinase B (AKT)/mTOR pathway. Additionally, metabolic enzymes influence macrophage polarization through epigenetic modifications that do not require transcription. Hexokinase 2 (HK2)-driven glycolysis induces lactate signaling, leading to histone lactylation, which further reinforces M1 gene expression in liver macrophages during metabolic disease (51).

#### 2.2.2 OXPHOS and FAO support M2 polarization

M2 macrophages, which possess the metabolic characteristics of anti-inflammation and tissue repair, depend heavily on OXPHOS and FAO to meet energy requirements and sustain their functional phenotype. This metabolic characteristic is the opposite of M1 macrophages, which are glycolytic-dependent and support proinflammatory responses. The OXPHOS and FAO pathways enable M2 macrophages to function as anti-inflammatory cells, promoting the resolution of inflammation.

Several studies have clarified the role of fatty acid metabolism in promoting M2 polarization. The fatty acid-binding protein 5 (FABP5) has been identified as a key regulator in macrophage alternative activation. Deletion of FABP5 in myeloid cells results in an accumulation of free long-chain unsaturated fatty acids such as oleic acid, which enhances fatty acid β-oxidation, the tricarboxylic acid (TCA) cycle, and OXPHOS, thereby encouraging M2 polarization through activation of the peroxisome proliferator-activated receptor gamma (PPARγ) signaling pathway (52). This highlights the essential role of lipid handling and mitochondrial metabolism in determining macrophage phenotypes. Similarly, in lung inflammation models, therapeutic interventions have been illustrated to suppress proinflammatory cytokines and promote M2 polarization by modulating fatty acid metabolism via the Akt signaling pathway. Treatment reduces fatty acid synthesis (FAS) and boosts FAO, leading to increased expression of M2 markers such as arginase-1 (Arg-1) and decreased iNOS expression (53). These metabolic shifts are mediated by downstream targets of Akt signaling, including ATP-citrate lyase (ACLY) and peroxisome proliferator-activated (PPARa), which regulate FAS and oxidation, respectively.

Several essential pathways regulate transcriptional metabolic reprogramming in M2 macrophages. Activation of PPAR family members, especially PPARγ and PPARα, triggers FAO and mitochondrial biogenesis gene programs. Additionally, signaling pathways such as the AMPK pathway enhance mitochondrial oxidative metabolism and FAO, further supporting M2 polarization (54). Other metabolic pathways involving mTOR and HIF-1α in target macrophages include the mTOR and HIF-1α pathways, which balance glycolysis and oxidative metabolism in response to environmental signals (55).

**Figure 3:**
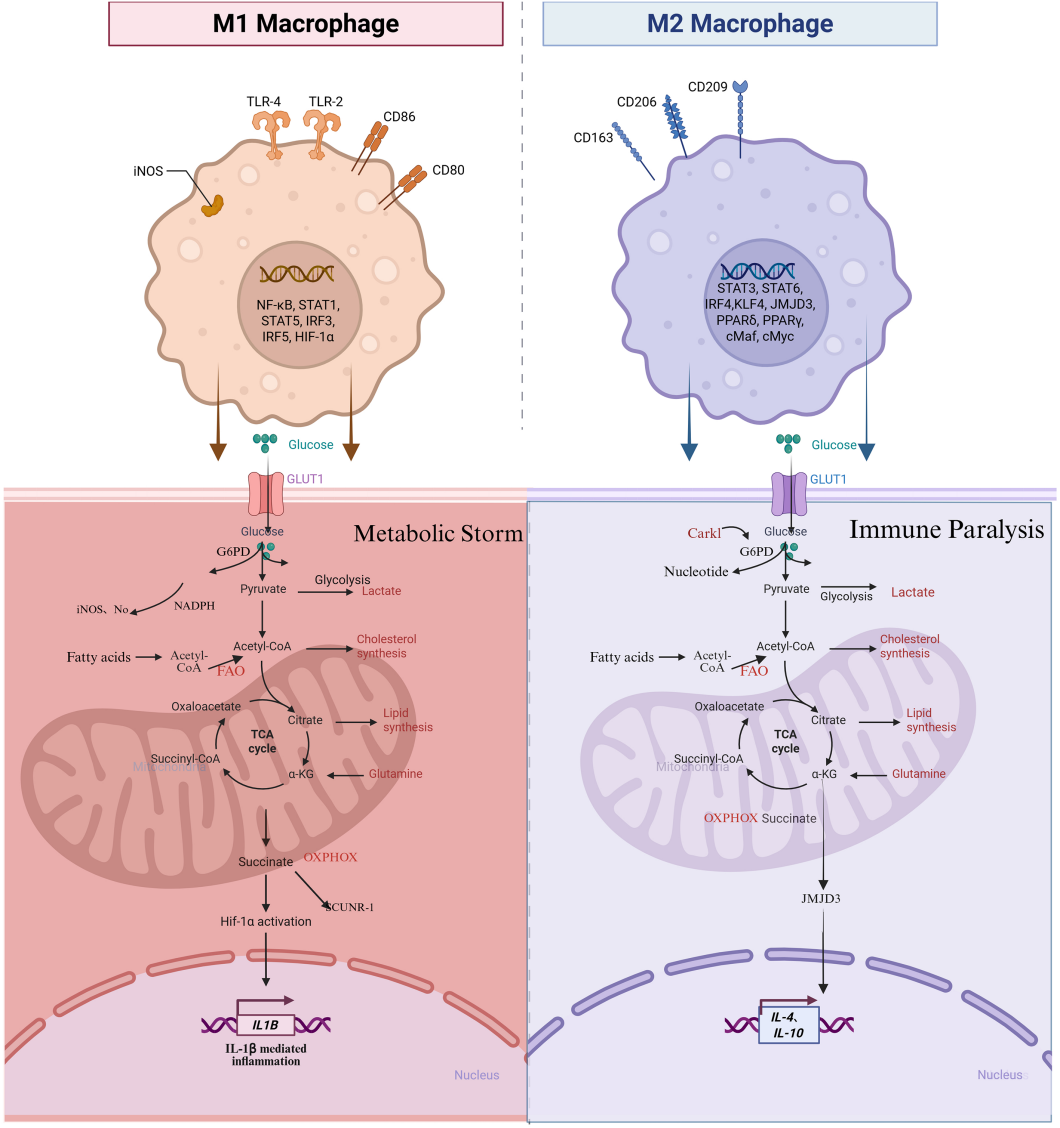
M1/M2 polarization imbalance and macrophage metabolic reprogramming (42). (Created with Biorender.com).

Additionally, mitochondrial chaperones, including heat shock protein 60, have been reported to maintain mitochondrial function and promote FAO in macrophages. The high expression of Heat Shock Protein 60 (HSP60) leads to FAO. Meanwhile, the activity of MRC complexes remains unchanged and is associated with low inflammation and restored metabolic health in disease models, including nonalcoholic fatty liver disease (NAFLD) (56). These studies conclude that mitochondrial quality control networks support the oxidative metabolic mode underlying M2 macrophage function, which is essential. M2 macrophages rely on oxidative metabolism in FAO and OXPHOS, facilitating tissue repair (**Figure 3**) (57, 58).

#### 2.2.3 Regulation of macrophage function by metabolic intermediates

**Figure 4:**
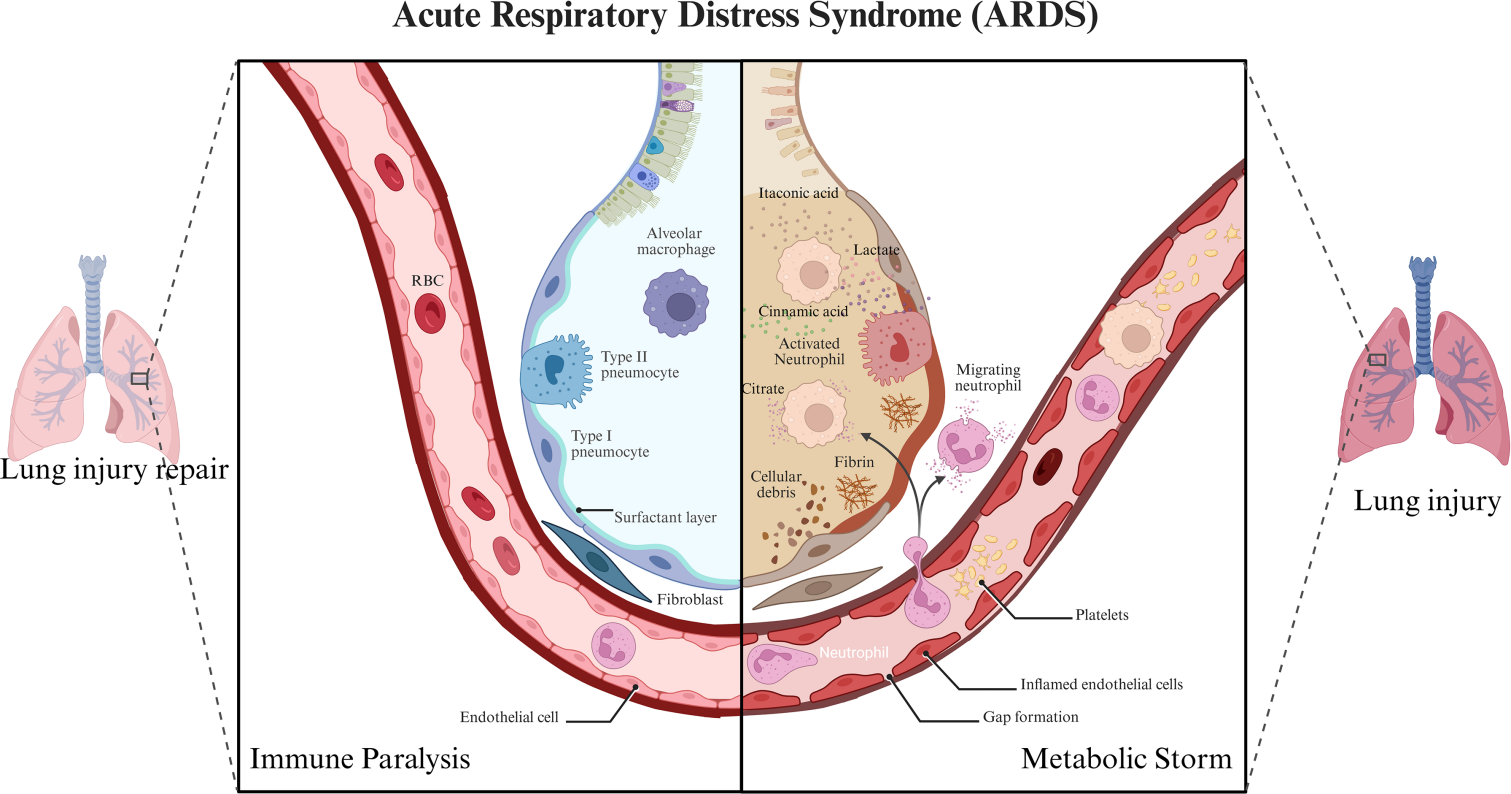
Regulation of macrophage function by metabolic intermediates in ARDS. (Created with Biorender.com).

Metabolic intermediates like lactate and citrate dynamically influence macrophage polarization and function by modulating inflammatory signaling pathways (**Figure 4**). First, lactate, which was traditionally seen as a metabolic byproduct of glycolysis, is now recognized as a powerful signaling molecule that determines macrophage phenotype. Adipocyte-secreted lactate acts as a danger signal. It promotes M1-like macrophage polarization by directly binding to prolyl hydroxylase domain-containing protein 2, thereby stabilizing HIF-1α and upregulating its downstream target, IL-1β, which contributes to systemic and adipose tissue inflammation (59, 60).

Citrate, a critical metabolite of the Tricarboxylic acid cycle (TAC), supports the biosynthesis of inflammatory mediators. The accumulation of citrate enhances prostaglandin and NO biosynthesis by activated macrophages, thereby increasing inflammatory signaling (61–63). Similarly, since succinate binds to succinate receptor 1 (SUCNR1) to act as a signaling metabolite, it influences macrophage development by inducing M2 hyperpolarization via the Gq signaling pathway (64). However, succinate-dependent macro-specific activity is context-dependent, with evidence supporting both enhanced anti-inflammatory and proinflammatory activity upon succinate binding, respectively (65)(**Figure 4**).

Citrate and lactate also intersect with lipid metabolism pathways to regulate macrophage polarization. Arachidonic acid metabolism, downstream of citrate, controls macrophage alternative activation by modulating OXPHOS via PPARγ, with prostaglandin E2 (PGE2) derived from arachidonic acid facilitating M2 polarization (66). Moreover, lipid metabolites such as leukotriene B4 (LTB4), produced via Arachidonate 5-lipoxygenase (ALOX5) from arachidonic acid, recruit M2 macrophages by activating the PI3K pathway, promoting tumor progression in intrahepatic cholangiocarcinoma (67). These findings highlight the complex interplay between metabolic intermediates and lipid-derived metabolites in shaping macrophage phenotypes.

Cinnamic acid facilitates the polarization of anti-inflammatory M2 macrophages, a process intricately linked to the activation of the STAT6 and PPARγ signaling pathways (68). Additionally, cinnamic acid may modulate the functional status and polarization trajectory of macrophages in sepsis by intervening in signaling pathways such as extracellular regulated protein kinases (ERK)1/2 (69). This metabolic reprogramming serves as the foundation for the transition of macrophage polarization states. Moreover, cis-aconitate can also orchestrate the shift in macrophage polarization states by adjusting histone acetylation levels and consequently impacting gene expression patterns.

Itaconic acid promotes antioxidant response by activating Nrf2 signaling pathway, further exerting its immune regulatory function. Nrf2 is the main antioxidant transcription factor in cells, capable of inducing the expression of various antioxidant enzymes and protecting cells from oxidative stress damage. Itaconic acid promotes the stabilization and nuclear translocation of Nrf2 through covalent modification with Keap1, enhancing glutathione synthesis, inhibiting ROS generation, reducing inflammatory factor release, and improving sepsis-induced tissue damage (70). In sepsis models, the accumulation of succinic acid and its induced HIF-1α stabilization are considered important factors in triggering excessive inflammatory responses (71). Through this mechanism, succinic acid promotes the expression of pro-inflammatory factors and the inflammatory cascade, leading to aggravated tissue damage. In sepsis and related inflammatory states, succinic acid promotes the chemotaxis and activation of macrophages through succinate receptor 1 (GPR91), exacerbating the inflammatory response (64).

**Figure 5:**
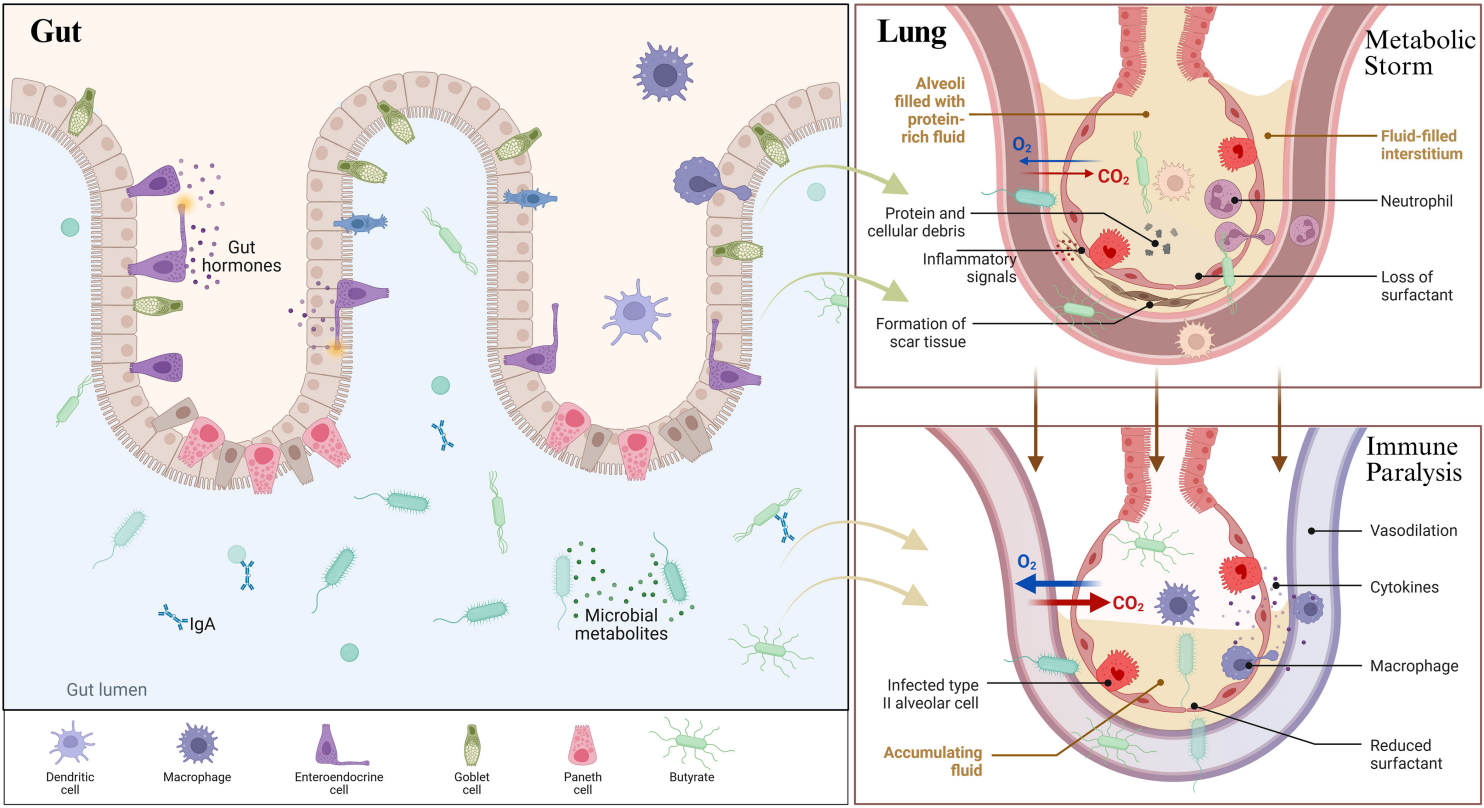
Regulation of macrophage function by gut metabolic intermediates. (Created with Biorender.com).

In addition to lactate and citrate, other metabolites such as butyrate — a short-chain fatty acid generated by gut microbiota shape macrophage polarization by stimulating the M2 phenotypes and quench the production of proinflammatory cytokines(**Figure 5**). Moreover, microbial metabolites, including reuterin, instruct tumor-associated macrophages to adopt an M1 antitumor phenotype through a reprogramming mechanism, thereby enhancing the efficacy of immunotherapy.

These metabolites modulate macrophage function through various signaling pathways, including stabilizing transcription factors, activating or inhibiting nuclear receptors, regulating kinase cascades, and inducing epigenetic modifications that influence gene expression. Lactate competitively inhibits α-ketoglutarate binding to prolyl hydroxylase (PHD2), leading to stabilization of HIF-1α and activation of proinflammatory macrophages. Itaconate exerts an anti-inflammatory effect by modulating the Kelch-like Epichlorohydrin (ECH)-ECH-associated protein (Keap1)-Nrf2 pathway and inhibiting the stimulator of interferon genes (STING) pathway, thereby reprogramming macrophage immune responses and metabolism (72).

### 2.3 Functional characteristics of macrophages in the “metabolic storm” phase

#### 2.3.1 Metabolic activity and enhanced inflammatory response

Within hours to days after the onset of sepsis, the condition typically progresses to a “metabolic storm” phase, marked by hyperactive immune cell metabolism and the release of a substantial amount of inflammatory factors, alongside systemic inflammatory response syndrome. During this stage, the host’s recognition of pathogen-associated molecular patterns (PAMPs) or damage-associated molecular patterns (DAMPs) activates the innate immune system, setting off a widespread and unrestrained immune response that culminates in a cytokine storm. This cytokine storm is characterized not only by the explosive release of pro-inflammatory cytokines such as TNF-α and IL-6 but also by aberrant metabolic changes, particularly the metabolic reprogramming of immune cells. M1 macrophages, a classic example of pro-inflammatory cells, exhibit metabolic reprogramming primarily characterized by enhanced glycolytic activity and disruption of the TCA cycle, accompanied by the accumulation of key metabolites like succinate and citrate. These metabolites not only function as intermediates in energy and material metabolism but also play a pivotal role by facilitating inflammatory signal transduction. Specifically, activated M1 macrophages demonstrate significantly elevated glycolysis, primarily driven by the upregulation of the glucose transporter 1 (GLUT1), key enzymes such as hexokinase 2, PFKFB3, and the glycolysis regulatory factor HIF-1α. These molecules collectively enhance glucose uptake and metabolism, thereby meeting the rapid energy demands of macrophages and facilitating the synthesis of inflammatory mediators (73). During the metabolic storm phase, M2 macrophages exhibit low-level expression of their markers, characterized by reduced expression of Arg-1band CD206, along with decreased activity of enzymes involved in FAO. Typically, M2 macrophages perform anti-inflammatory and tissue-repairing functions, and their activity hinges on the activation of metabolic pathways such as FAO.

Furthermore, the synthesis of other metabolic intermediates, such as succinate, might stabilize HIF-1α, a potent inducer of glycolysis and inflammatory cytokine production, thereby increasing its localization and further activating the inflammatory loop (73). This metabolic crisis in macrophages leads to progressive tissue damage by fueling inflammation and hindering resolution. Sequestered mitochondria and accumulated ROS, inherent in sepsis, are produced by macrophages and present a detailed pattern of defective clearance of dysregulated cells and Alveolar destruction. These conditions are correlated with poor outcomes in ARDS patients (74). Increased glycolysis and inflammasome-dependent upregulation of inflammasome NLRP3, involved in the maturation and release of IL-1β, an essential cytokine of sepsis-induced inflammation, are enhanced by mitochondrial inhibition (75–78).

#### 2.3.2 Activation of metabolic regulatory networks

Metabolic regulatory networks in macrophages during sepsis-induced ARDS are orchestrated by a complex interplay between signaling pathways and metabolic enzymes, ultimately driving the inflammatory response and macrophage polarization. HIF-1α promotes the transcription of glycolytic enzymes and glucose transporters, thus implementing a metabolic switch from OXPHOS to aerobic glycolysis that supports the energetic and biosynthetic requirements of classically activated M1-polarized macrophages. The findings from experimental models demonstrated that HIF-1α knockout or inactivation reduces glycolytic flux, inhibits the production of proinflammatory cytokines, and diminishes lung lesions in LPS-induced ARDS, thereby highlighting the importance of HIF-1α in aligning metabolism with inflammation (12, 79).

Furthermore, several metabolic enzymes act as key effectors downstream of these signaling pathways. Lactate dehydrogenase A, which converts pyruvate into lactate, is one of the enzymes overexpressed during glycolytic activity in inflammatory macrophages. LDHA is phosphorylated and activated, promoting NF-κB signaling through increased ROS production in a positive feedback loop that sustains inflammation in sepsis-induced ARDS lung epithelial cells (80). Kaempferol can reduce septic ARDS inflammation by inhibiting the HIF-1α, NF-κB, and PI3K-Akt pathways in alveolar macrophages, thereby decreasing inflammation and apoptosis. The interconnected relationship between inflammation and metabolic signaling may alter both pathways, potentially offering a strategy to redirect macrophage-driven inflammation in sepsis-induced lung injury.

Dysregulation of glucocorticoid metabolism in alveolar macrophages, especially reduced activity of 11β-hydroxysteroid dehydrogenase type 1 reductase (HSD-1), has been linked to increased inflammation by diminishing the anti-inflammatory effects of local glucocorticoids. Impaired HSD-1 correlates with heightened NF-κB activation and poor clinical outcomes in sepsis-induced ARDS, underscoring the significance of metabolic enzyme activity in both pro- and anti-inflammatory signaling. Recent research also highlights the role of aquaporins, particularly aquaglyceroporins like aquaporin 3 (AQP3) and aquaporin 9 (AQP9), in transporting metabolic substrates, such as glycerol, which stimulates glycolysis and energy production in immune cells. AQPs also influence key inflammatory pathways, including NF-κB and toll-like receptor 4 (TLR4), as well as the functions of macrophages and neutrophils during sepsis (81). Pharmacological targeting of AQPs has demonstrated promising results in sepsis models by reducing inflammation and organ damage, further emphasizing the role of metabolism within the immune system.

#### 2.3.3 Contribution of metabolic storm to lung tissue injury

Metabolic storm, characterized by profound metabolic reprogramming and dysregulation, plays a pivotal role in exacerbating lung tissue injury during sepsis-associated ARDS (82, 83). Hyperglycemia promotes SARS-CoV-2 infection and monocyte activation via an HIF-1α-dependent mechanism on glycolysis, resulting in mitochondrial ROS and HIF-1α stabilization. This metabolic reprogramming not only promotes viral replication but also inhibits T cell responses and compromises epithelial cell survival, contributing to lung injury (84). Interactions with inflammatory cytokines, such as interleukin-6, exacerbate lung damage. IL-6 enhances ferroptosis in alveolar epithelial cell (AEC-II) by affecting bile acid metabolism and the farnesoid X receptor (FXR), which is critical for lung regeneration. FXR inhibition alleviates IL-6-induced ferroptosis and inflammation, as observed by cell death and 5-Ethynyl-2’-deoxyuridine (EdU) staining assays. Together, these findings highlight the crucial role of metabolic reprogramming in regulating cell death and tissue repair (85). Mitochondrial dysfunction is another hallmark of metabolic storm-induced lung injury, as demonstrated by a study of lung epithelial cells. Pallmitoylation of Cyclooxygenase-2 (COX-2) at Cys555 increases COX-2’s interaction with HK2, driving mitochondrial metabolic reprogramming and, thus, the proteolytic inflammatory injury pyroptosis via activation of the NLRP3 inflammasome in mitochondria. This same study demonstrates that palmitoylation inhibition of hexokinase 2 (HK2) at Cys555 decreases metabolic and lung injury associated with inflammation (86–88).

Simultaneously, metabolic reprogramming causes endothelial dysfunction to worsen, leading to lung injury. Growth differentiation factor 15 (GDF15) protects against sepsis-induced ALI by suppressing the HIF-1α/LDHA glycolytic axis in pulmonary ECs, thereby reducing cytokine production and leukocyte recruitment. As a result, the loss of this regulation enhances inflammation and barrier dysfunction, contributing to alveolar damage. Overall, metabolic disturbances during sepsis lead to oxidative stress, ferroptosis, pyroptosis, and apoptosis in ECs (89). Abnormalities in metabolic reprogramming—including uncontrolled glycolysis, mitochondrial dysregulation, and specific signaling pathways like HIF-1α, IGF1R, and FXR—initiate these processes. Cell injury then compromises the alveolar-capillary barrier, worsens inflammation, and impairs lung regeneration, collectively promoting tissue damage. Modulating metabolic reprogramming, including targeting glycolytic enzymes, mitochondria, and metabolic receptors, may help reduce lung injury in SAARD and improve patient outcomes (83–89).

### 2.4 Metabolic suppression and macrophage dysfunction in the “immunoparalysis” stage

#### 2.4.1 Inhibition of energy metabolic pathways leading to immune dysfunction decline

On the 3rd to 7th day of sepsis progression, the immune system enters a paralysis phase, characterized by a marked reduction in the expression of pro-inflammatory factors in M1 macrophages, as evidenced by decreased secretion of TNF-α, IL-1β, and other cytokines. Concurrently, immunosuppressive molecules such as programmed death ligand 1 (PD-L1) and the anti-inflammatory cytokine IL-10 exhibit upregulated expression. This alteration in expression patterns signifies a transition of the immune system from a hyperactivated to a suppressed state, resulting in compromised host immune function and an elevated risk of infection. Additionally, the activity of glycolysis-related enzymes declines, metabolic processes slow down, and the energy supply to M1 macrophages becomes restricted, further impairing their pro-inflammatory capabilities. Multiple signaling pathways are implicated in this regulatory mechanism. Androgen receptor (AR) signaling enhances M1 polarization and pro-inflammatory factor secretion by upregulating FKBP prolyl isomerase 5 gene (FKBP5) expression and activating the NF-κB pathway, whereas AR deficiency inhibits M1 polarization and mitigates lung injury. Moreover, Nrf2 deficiency can intensify M1 polarization and inflammation, while Nrf2 plays a protective role by modulating autophagy and the PPARγ/NF-κB pathway to maintain a balance between M1 and M2 polarization (90).

Mitochondrial OXPHOS also generates much energy and ROS that kill oxygen-dependent pathogens. However, in sepsis-induced ARDS, this metabolic pluripotency appears to be pushed to the background. Studies have confirmed that glycolytic function and mitochondrial respiration are both reduced in macrophages throughout immune paralysis. This results in not only a lack of ATP supply but also cellular impairment (91).

The energy deficit results in a reduced ability for phagocytosis and impeded secretion of pro-inflammatory cytokines, such as TNF-α, IL-1β, and IL-6, all of which are defining features of immunosuppression. Glycolytic enzymes’ downregulation and mitochondrial dysfunction are involved in this metabolic deficiency. The inhibition of crucial glycolytic enzymes or mitochondrial complexes prevents the generation of enough energy and biosynthetic precursors that activate and support a macrophage effector response (92). Mechanistically, the suppression of metabolic pathways in macrophages during sepsis may be linked to signaling abnormalities. The latter includes low activation levels of AMP-activated protein kinase and the mammalian target of rapamycin, which maintain both immunity and cellular energy homeostasis (92).

Immunoparalysis of macrophages is further metabolically linked to tissue microenvironment-specific mitochondrial crosstalk. The harsh acidity, notably extracellular lactate or other metabolites present in the microenvironment of inflamed tissues, represses macrophage glycolysis and OXPHOS; therefore, it suppresses the function of macrophages (93). Furthermore, the overabundance of immune-assassin metabolites, including methylmalonic acid, can contribute to systemic metabolic dysfunction and exhaustion in CD8+ T cells, suggesting that metabolic disorders may have broader implications for immune activities beyond macrophages (94). Importantly, interventions targeting metabolic pathways have demonstrated promise in reversing immune dysfunction. Pharmacological activation of AMPK or inhibition of mTOR can restore metabolic activity and improve macrophage function, enhancing phagocytosis and cytokine production (93). Nanomedicine strategies to modulate metabolic processes in immune cells, such as glycolysis or mitochondrial improvements, have the potential to reverse metabolic processes associated with immune competence (95, 96).

#### 2.4.2 Changes in macrophage polarization under immunosuppressive conditions

**Figure 6:**
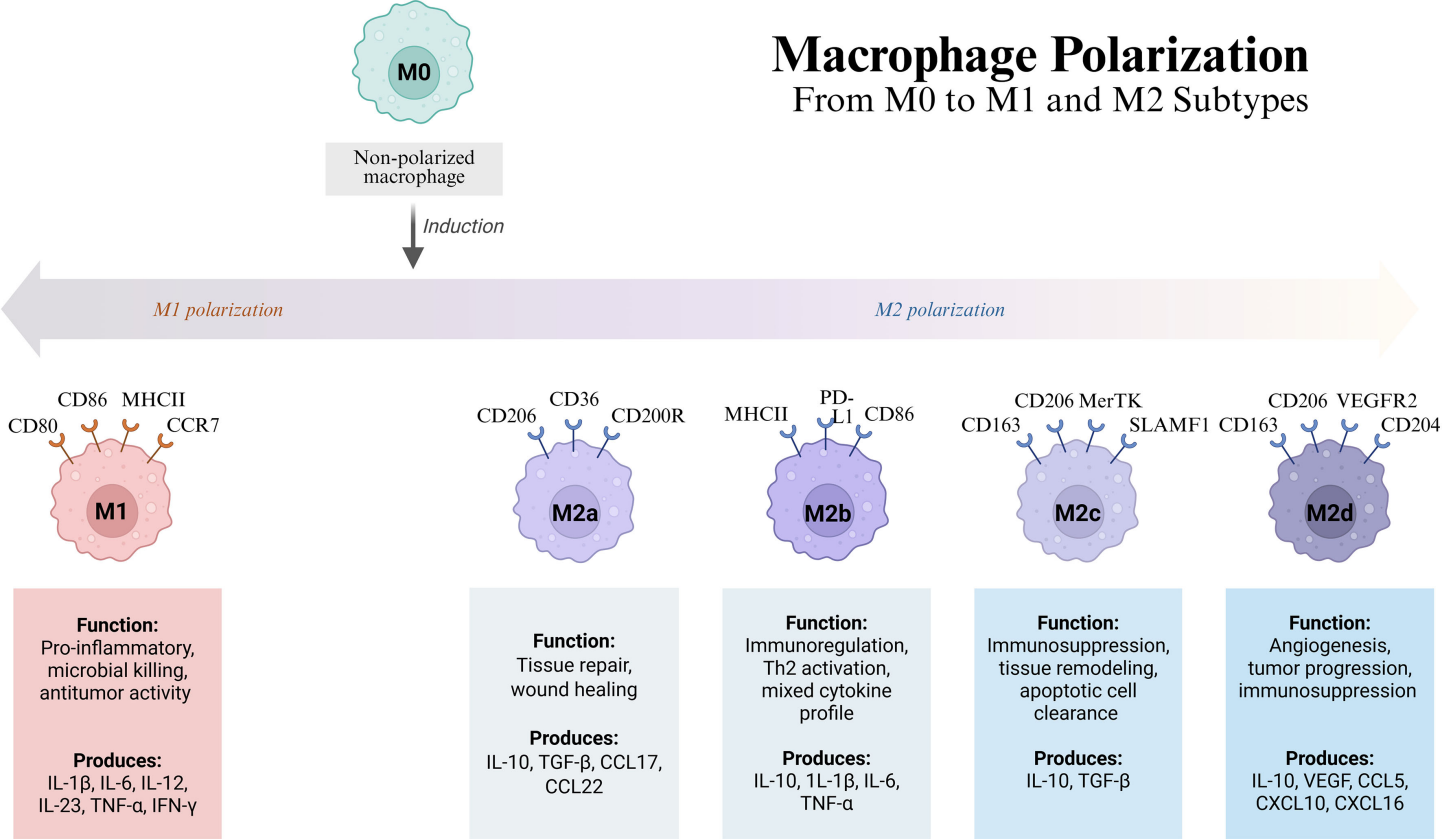
Macrophage polarization and M2 phenotype. (Created with Biorender.com).

The increased M2 polarization phenotype observed in macrophages under immunosuppression suggests a bias toward a more anti-inflammatory, tissue-repair-oriented phenotype. Nonetheless, at the expense of an enhanced M2 orientation, the functional capacity of these macrophages is diminished, a state that skews immune regulation in an imbalanced manner, potentially promoting pathological conditions rather than ameliorating them (**Figure 6**). This phenomenon has been observed in the context of sepsis-associated ARDS and tumor microenvironments, and is well described in several studies. This clearly indicates that immunosuppression can negatively affect macrophage activity, regardless of the polarization state (97, 98). Furthermore, lymphatic leakage fosters an immunoregulatory environment by promoting M2 via medical irradiation, highlighting the essential role of the tissue microenvironment in macrophage polarization (99, 100).

Anti-inflammatory cytokines, growth factors such as VEGF and PDGF, and matrix metalloproteinases secreted by immunosuppressive M2 phenotype macrophages promote tumor growth, metastasis, and immune escape. This phenotype recruits and supports other immunosuppressive cell types, such as regulatory T cells and B cells, as well as alternatively activated neutrophils, collectively forming a complex immunosuppressive niche (101–106).

Moreover, emerging evidence has highlighted the contribution of KO of macrophage polarization to metabolic reprogramming (107, 108). The M2 macrophage subtypes (M2a, M2b, M2c, M2d) exhibit diverse immunosuppressive and pro-angiogenic functions, secreting factors that promote tumor progression and modulate the activity of other immune cells to maintain an immunosuppressive microenvironment (101). Glioma stem cells secrete Follistatin-like 1(FSTL-1), which sustains stemness and induces M2 polarization via TLR2-mediated PI3K-AKT signaling, highlighting tumor-stroma crosstalk in immunosuppression (109).

Targeting metabolic enzymes and pathways holds therapeutic potential for reversing M2 polarization. Inhibition of Pim2 kinase reduces glycolytic reprogramming and M1 polarization in inflammatory arthritis. At the same time, nuclear phosphoglycerate dehydrogenase (PHGDH) regulates macrophage polarization via transcriptional repression of glutamine metabolism enzymes in breast cancer, promoting immunosuppressive M2 phenotypes (110, 111). Additionally, mitochondrial metabolism and FAO have been implicated in maintaining M2 polarization, with interventions that modulate these pathways shifting macrophages toward anti-tumor phenotypes (112–114).

#### 2.4.3 Impact of immune paralysis on the prognosis of sepsis-associated ARDS

Immune paralysis, also known as immunosuppression, represents a major driver of adverse outcomes in ARDS associated with sepsis. In the late phase of sepsis, the previously hyperinflammatory state transitions into a state of profound immune dysfunction, characterized by the suppression of both innate and adaptive immune responses. Maladaptive changes in macrophage metabolism in sepsis contribute to a decrease in phagocytic and antigen-presenting activity, leading to immune paralysis. Whereas metabolic reprogramming involving the upregulated glycolysis and impaired mitochondrial bioenergetics promotes M1 polarization in the early phase of the septic insult, in the later course of the disease, it leads to macrophage exhaustion and reduced antimicrobial capacity, which facilitates the development of secondary infections and prolonged inflammatory-mediated lung injury (115, 116).

During the immunosuppressive phase, the amounts of pro-inflammatory cytokines produced drop, and the up-regulation, combined with the activation of NLRP3 inflammasomes, is impaired. Factors, including metabolic regulators such as HIF-1α and enzymes like Succinate Dehydrogenase (SDH), altered macrophage responses and generated a switch in their power status, affecting the pattern between immune activation and paralysis (117).

Furthermore, the immune dysfunction in immune paralysis allows recurrent infections and continuous lung tissue injury, thereby extending the disease course. The inability to properly eradicate the pathogens leads to chronic alveolar damage, increased vascular permeability, and worsened ARDS pathophysiology. There is evidence that macrophage metabolic dysregulation, such as reduced OXPHOS and increased ROS production, also induces mitochondrial damage and promotes cell death. Therefore, immune defense in the lung microenvironment becomes crippled (118, 119). This process forms the oxygen in a cycle of immune dysfunction and lung tissue injury. This highlights the need to normalize macrophage metabolism to improve the course of ARDS in sepsis.

Metabolic reprogramming of other innate immune cells, namely neutrophils and dendritic cells, during sepsis also results in an extreme immunosuppressive environment. The decrease in glycolysis and FAO in these immune cells impairs their response to infections, leading to increased cytokine production and increased susceptibility to secondary infections (120, 121). Immune paralysis brings about systemic immune depression that results in not only extension of mechanical ventilation and intensive care unit timeframe, but also high susceptibility to hospital-acquired infections, which are challenging to manage and lead to an increased possibility of a patient’s death. The recent study suggests that metabolism-based therapies can reverse the above-described process. These include agents such as metformin and itaconate derivatives, which are capable of reprogramming the macrophages’ metabolism, thereby helping to decrease excess inflammation and increase bacterial clearance capacity to alleviate lung injury in sepsis models, as well as improving the chances of survival (116). Furthermore, targeting key metabolic enzymes, such as the muscle-type 6-phosphofructokinase (PFKM), to inhibit glycolysis has demonstrated protective effects against sepsis-induced cytokine storms and organ damage (122–125).

### 2.5 BMSC-EXO in regulating macrophage metabolism

#### 2.5.1 Immunomodulatory Functions of BMSC-EXO

BMSCs secrete exosomes that carry a diverse array of bioactive molecules, including microRNAs (miRNAs), proteins, and signaling lipids, which collectively contribute to their potent immunomodulatory effects. These exosomes have been demonstrated to regulate inflammatory responses and modulate immune cell functions across various disease models, underscoring their therapeutic potential. BMSC-derived exosomes (BMSC-EXO) deliver the protein JNK activating protein (JKAP) to restore the balance between pro-inflammatory Th17 cells and anti-inflammatory regulatory T cells (Tregs) by activating AKT/ERK signaling pathways, thereby alleviating disease symptoms and inflammation (126–128).

Beyond T cell modulation, BMSC-EXO also affect the functions of the innate immune cells, including macrophages and dendritic cells (129). In sepsis-induced lung injury, BMSC-EXO serum amyloid A1 (SAA1) reduces inflammation and endotoxin levels, promoting alveolar macrophage function and attenuating lung damage (130). These findings underscore the capacity of BMSC-EXO to modulate both adaptive and innate immune responses by delivering specific proteins and miRNAs that regulate key signaling pathways, including the NF-κB, NLRP3 inflammasome, and cytokine networks (131–133). These data collectively highlight the broad anti-inflammatory and immunoregulatory actions of BMSC-EXO, which are mediated through the modulation of inflammasome activation, cytokine secretion, and immune cell polarization (134). This suggests that environmental cues modulate the cargo and functional potency of BMSC-EXO, enhancing their immunoregulatory capabilities.

#### 2.5.2 BMSCs-derived exosomes regulate macrophage polarization by inhibiting glycolysis

BMSC-EXO is rich in miRNAs, which can target and modulate key molecules within macrophages, thereby facilitating the transition from M1 to M2 polarization. Studies have revealed that miR-23a-3p, highly expressed in BMSC-EXO, can specifically target and inhibit interferon regulatory factor 1 (IRF1), a pivotal regulatory factor for M1 polarization (135). Moreover, BMSC-EXO overexpressing miR-23a-3p significantly promotes the polarization shift of macrophages from the M1 to the M2 phenotype. miR-25-3p suppresses M1 polarization and fosters M2 polarization by inhibiting the activity of the JAK2/STAT3 signaling pathway. These miRNAs orchestrate metabolic reprogramming and functional transformation in macrophages through precise modulation of inflammation-related signaling pathways (136). Additionally, BMSC-EXO carries specific proteins that also play a role in regulating macrophage polarization. Research indicates that the CISH protein delivered by BMSC-EXO can inhibit the activation of the NF-κB signaling pathway, thereby promoting macrophage polarization towards the M2 phenotype (137).

BMSC-EXO inhibits glycolysis by downregulating the expression of key glycolytic enzymes. It significantly reduces the expression of enzymes closely associated with glycolysis in macrophages, including hexokinase (HK), phosphofructokinase (PFK), and lactate dehydrogenase (LDH). Elevated activity of these enzymes drives macrophage metabolism towards glycolysis, resulting in the production of substantial amounts of lactic acid and supporting the M1 polarization phenotype. The intervention of BMSC-EXO diminishes the activity of the glycolytic metabolic pathway by lowering the stability and expression of HIF-1α, consequently reducing the expression levels of the aforementioned enzymes. This, in turn, suppresses the release of pro-inflammatory cytokines such as TNF-α and IL-6, effectively mitigating inflammatory injury and pathological alterations in lung tissue. By inhibiting the phosphorylation of p65 and p38, BMSC-EXO reduces the proportion of M1-polarized macrophages. It can also inhibit the glycolytic activity of macrophages, diminish the expression and activity of key glycolytic enzymes, and lower lactate production, thereby attenuating the metabolic characteristics of pro-inflammatory M1 macrophages. Concurrently, BMSC-EXO promotes the recovery of mitochondrial function in macrophages, elevates the level of oxidative phosphorylation, increases ATP production, and enhances the activity of metabolic pathways such as FAO, thereby facilitating the polarization of macrophages towards the M2 phenotype.

BMSCs have become potent modulators of the immune response in sepsis-induced ARDS, particularly through exosomal secretion that influences macrophage activity. A crucial mechanism by which BMSC-EXO target and cure is by inhibiting glycolysis in macrophages, thereby altering the regulatory state of these cells. Macrophages modify their metabolism during inflammation, with the M1 phenotype upregulating glycolysis through the HIF-1α pathway, thereby enhancing the regulation of glycolytic genes. This metabolic transition is expected to support the pro-inflammatory target, thereby precipitating lung damage in ARDS. Recent studies have demonstrated that exosomes secreted by BMSCs can downregulate HIF-1α expression in macrophages, leading to decreased activity of key glycolytic enzymes and a subsequent reduction in glycolytic flux. *In vitro* analysis using murine alveolar cell line MH-S cells primed with LPS showed that BMSCs’ exosomes diminished M1 and promoted M2 markers, which concurred with reduced glycolysis.

#### 2.5.3 Therapeutic potential of BMSC-EXO in LPS-induced ARDS models

BMSC-EXO as a potential therapeutic agent for the treatment of ARDS. This approach is particularly suited to LPS-induced ARDS models, which are commonly used to study sepsis-related lung injury. Experimentally, BMSC-EXO have been illustrated to markedly reduce inflammation and alleviate pathological injury in pulmonary tissues, thereby enhancing lung function. Macrophage polarization is a critical element. M1/M2 amacrophage transition deficiency has devastating effects on the amacrophage. Although multiple biological functions can contribute to this situation, macrophage polarization is the most likely cause, as the LPS-induced switch is designed to increase M1 and reduce M2 inflammatory macrophages. Metabolism is the BMSC, while M1 inflammation has several elaborate metabolic processes.

BMSC-EXO have been demonstrated to carry microRNAs that regulate key cellular processes in alveolar macrophages. A following critical microRNA was miR-384-5p, the levels of which in BMSC-EXO were markedly high. Meanwhile, unlike miR-2909, exosomal miR-384-5p increased, rather than decreased, cell viability. MicroRNA activation occurs not through pyroptosis but by targeting the gene Beclin-1, which plays a critical regulatory role in autophagy. BMSC-EXO are described to alleviate autophagy dysfunction in phagocytes, inhibit apoptosis, and reduce HeLa cell second death. This overall mechanism contributes to the reduction of lung inflammation, the restoration of vascular permeability, and ultimately, to improved survival when BMSC-EXO are administered to LPS-induced ALI rats via intravenous or intratracheal routes (138). *In vivo*, hBMMSC-exo reduced lung injury scores, decreased inflammation and cell infiltration, and protein leakage in bronchoalveolar lavage fluid, and enhanced survival in septic animals. To our knowledge, this study is the first to show the therapeutic power of BMSC-EXO in modulating macrophage metabolism and inflammation in ARDS. However, the therapeutic potential of MSCs depends primarily on the source of MSCs (139).

Beyond bacterial endotoxin-induced injury, BMSC-EXO have demonstrated efficacy in viral pneumonia models, such as murine cytomegalovirus (CMV)-induced pneumonia. In this context, exosome-treated mice showed a shift in MMSC from an inflammatory M1 to a reparative M2 phenotype, with reduced infiltration of inflammatory cells and improved pulmonary fibrosis. This anti-inflammatory and anti-fibrotic effect of exosomes was due to the downregulation of the NF-κB/NLRP3 inflammasome signaling pathway. We can suggest that the inhibition of macrophage-driven inflammation and fibrotic remodeling may explain how BMSC-EXO attenuate experimental endotoxin-induced ARDS and hold therapeutic potential for patients with viral-induced lung injury (140).

### 2.6 Clinical significance and future directions of macrophage metabolic reprogramming in sepsis-induced ARDS

#### 2.6.1 Potential of metabolic reprogramming as diagnostic and prognostic biomarkers

Metabolic reprogramming has been identified as a fundamental hallmark in a broad spectrum of diseases. As a result, metabolic reprogramming is a promising biomarker for diagnostic and prognostic applications (141–146). In this regard, metabolic pathways of macrophages, especially glycolysis and OXPHOS, which represent the two polarization states of macrophages, M1 proinflammatory and M2 anti-inflammatory, vary according to the tissue disease gradient and dominate over each other. The dynamic metabolic state of macrophages, encompassing glycolysis, lipid metabolism, and amino acid metabolism, is linked to immune responses, organ failure, and reparative responses in sepsis and COVID-19 (147, 148).

Macrophage polarization is directly linked to metabolic reprogramming. Nowadays, metabolic reprogramming is considered an additional metabolic signature of disease and a marker of responsiveness to existing treatments. In this context, macrophage polarization depends on their glycolytic flux, FAO and amino acid metabolism. At the same time, their polarization has implications for the profile of secreted cytokines. It serves as the basis for the current development of innovative treatments utilizing macrophages in patients with sepsis, cancer, or chronic inflammation (149–152). Moreover, in sepsis: pathogen-induced lung injury (153), metabolic biomarkers of macrophage polarization state are associated with disease severity and therapeutic response to lung-protective mechanical ventilation (153).

The advent of multi-omics technologies, including single-cell RNA sequencing, metabolomics, and proteogenomics, has enabled the identification of highly sensitive and specific biomarkers associated with metabolic reprogramming (154, 155). Significantly, metabolic biomarkers not only indicate the state of disease but also immediately suggest the underlying causes and mechanisms of therapy resistance (156, 157). In sepsis, metabolic shifts in macrophages contribute to immune paralysis and organ dysfunction (158, 159). These insights enable the development of targeted interventions that modulate metabolic pathways to restore immune function and improve clinical outcomes. Metabolism-related molecules and macrophage polarization states represent promising diagnostic and prognostic biomarkers across diverse diseases, including sepsis-induced ARDS. Their dynamic nature allows real-time monitoring of disease progression and therapeutic efficacy.

#### 2.6.2 Targeted therapeutic strategies based on metabolic regulation

Targeting macrophage metabolism has emerged as a promising therapeutic avenue in sepsis-associated ARDS, aiming to restore immune homeostasis by fine-tuning inflammatory responses through metabolic reprogramming. Macrophages, crucial innate immune cells during sepsis and ARDS, shift their metabolism from OXPHOS to glycolysis to meet the increased energy demands of inflammation. Additionally, discovering metabolic advances might sustain the plastic induction of OXPHOS-dependent converting M1.

Compounds such as the natural molecule kaempferol have been effective in treating sepsis-induced ARDS by targeting critical metabolic and inflammatory signaling pathways. As demonstrated by network pharmacology and preclinical-based studies, kaempferol downregulates key pathways, including the HIF-1a, NF-κB, and PI3K-Akt pathways. As a result, proinflammatory cytokines TNF-α, IL-1β, and IL-6 were drastically reduced. In addition to ROS suppression, apoptosis declined in alveolar macrophages. At this point, transcriptional inhibitors of these metabolic pathways that can resolve diseases by restoring immune homeostasis must be activated.

Crucial nodes for intervention are metabolic intermediates and the enzymes that catalyze their reactions. The SphK1/S1PR3 axis promotes glycolysis by upregulating HIF-1α and glycolytic enzymes HK2 and PFKFB3, and it also orchestrates sepsis-induced macrophage polarization into proinflammatory subsets. Pharmacological inhibition of sphingosine kinase 1(SphK1) with PF-543 rebalances macrophage phenotypes by attenuating glycolysis, inhibiting NF-κB activation, and protecting against pathogenic multi-organ damage (29). Closely related to this, enhancement of Spns2/S1P signaling represents a potential approach to neutralize both early hyperinflammation and late-stage immunosuppression by modulating macrophage metabolism and ROS (123). These findings underscore the therapeutic potential of dynamically modulating metabolic regulators to control macrophage function during sepsis.

Itaconate and its derivatives are illustrative of emerging metabolic regulators that contribute as endogenous immunometabolic checkpoints. Itaconate effectively inhibits succinate dehydrogenase, activates the in antioxidant pathway, and modulates glycolytic flux to coordinate the reduction of inflammatory output and oxidative stress in macrophages. Mice models and other preclinical studies have illustrated that itaconate derivatives can reduce cytokine storms and organ injury during sepsis, providing a basis for considering itaconate as a metabolic immunotherapy candidate (160). Moreover, Itaconate derivatives were combined with glycolysis inhibitors in nanoparticle formulations to serve as macrophage-reprogramming interventions, designed to improve the survival of mice with precipitated sepsis. This approach offers a new and creatively engineered modality for metabolic modulation of cells (72).

Therefore, AQPs, and more specifically aquaglyceroporins such as AQP3, AQP7, and AQP9, regulate macrophage metabolism by promoting glycerol transport, which is required for glycolysis and lipid metabolism for immune activation. In addition, AQP targeting also reduces macrophage inflammatory responses and neutrophil unregulated migration *in vivo*, as evident by reduced inflammation and lung injury in sepsis with AQP9 and AQP5 inhibitors. This renders metabolic substrate transporters attractive hypothetical therapeutic targets for immune cells and inflammation.

Metabolic reprogramming-mediated epigenetic regulation also serves as a therapeutic avenue. Methyltransferase-like 3 (METTL3)-mediated N6-methylation of adenosine in Trim59 mRNA protects against sepsis-induced ARDS by repressing NF-κB signaling and attenuating inflammatory responses. Therefore, targeting the metabolic-epigenetic link can reprogram the function of macrophages and ECs in ARDS (161).

Nanomedicine approaches further enhance metabolic targeting. The “nano-windmill,” a tetrahedral framework nucleic acid-based siRNA delivery system, suppresses macrophage activation by reducing choline uptake, thereby inhibiting glycolysis and inflammatory responses in sepsis models (162). Such precision delivery systems enable targeted modulation of specific metabolic pathways, minimizing off-target effects.

Nanomedicine makes metabolic targeting more sophisticated. A nano-windmill, a tetrahedral framework nucleic acid-based siRNA delivery system, reduces macrophage activation by inhibiting choline uptake, thus preventing glycolysis and inflammation in sepsis models (163). Such targeted delivery technology enables the regulation of metabolic pathways specifically in target cells, thereby preventing off-target side effects. Mitochondrial metabolism plays a quintessential role in macrophage function and is an important intervention target. Promoting mitochondrial biogenesis, proteostasis, and function can endow macrophages with the ability to restore homeostasis and thereby lessen organ injury. Transplantation of exogenous mitochondria has demonstrated an anti-inflammatory and anti-apoptotic effect in ARDS models. Moreover, selenium nanoparticle-modified MSCs facilitate mitochondrial transfer into alveolar epithelial cells and suppress the retinoic acid receptor-related orphan receptor gamma-t (RORγt)/STAT3/Th17 axis in sepsis, thereby reducing inflammatory lung injury (164, 165). Therefore, manipulating lipid metabolism may help restore immunity. Metabolic regulation-based therapeutic modalities targeting macrophages include inhibiting glycolysis and proinflammatory metabolic pathways, enhancing mitochondrial activity, modulating metabolic intermediates and transporters, employing epigenetic measures, and delivering precision nanomedicine. Those strategies allow reprogramming of macrophage polarization, attenuating cytokine storms, decreasing oxidative damage, and restoring immune competence. The heterogeneous and dynamic nature of sepsis and ARDS necessitates a dedicated, personalized approach to multi-targeted metabolism therapies that can achieve a balance between inflammation dampening and immune restoration.

#### 2.6.3 Future research directions and challenges

The intricate crosstalk between metabolic pathways and immune responses in sepsis-induced ARDS represents a critical frontier for future research, with the potential to revolutionize therapeutic approaches. As a result, macrophages, a leading orchestrator of the immune system, rapidly reprogram their metabolism in response to signals, thereby directing their polarization and function. Classical M1-like, proinflammatory macrophages rely on glycolysis for a significant proportion of their fuel, while anti-inflammatory M2-like macrophages prefer OXPHOS and FAO. The imbalances within this structure may exacerbate inflammatory tissue injury or diminish protective mechanisms, increasing the need to unravel these complex networks for medical benefit. M2-like macrophages were transferred to the lungs of mouse models of LPS-induced ALI using exosomes in the BMMSCs (166). The outcomes emphasize immunometabolic treatment, which is effective at the preclinical level and shows responsiveness in signaling pathways; several Mars depend on activation and its transmission, such as HIF-1α, NF-κB, and PI3K-Akt.

Moreover, the metabolic crosstalk is not limited to glycolysis, as the amino acid transporter and utilization also play a role in glutamate transport, which has a protective role in acid-base equilibrium and in reducing oxidative stress in sepsis-induced lung injury, an example of such interaction in cancer that supports a potential approach to use metabolic transporters and enzymes as treatment targets. Simultaneously, the role of aquaporins, especially AQP3 and AQP7, in the transport of glycerol and other small molecules essential for glycolysis and lipid synthesis has also been identified. This involves facilitating AQP3 and AQP7 in regulating macrophage energy demand, regulating inflammatory factor-dependent signaling via TLR4 via the energy-signaling-regulating kinase PI3K, and an excitatory role in macrophage-driven physiological changes. Sepsis treatment by modifying AQPs is efficient, reducing both organ damage and impairments in cellular immune function.

Nevertheless, many obstacles remain. Due to the complex nature of the dynamic sepsis setting, macrophage phenotypes and metabolic states are heterogeneous, making it challenging to search for common therapeutic targets. Moreover, bidirectional interactions between metabolic pathways and immune signaling networks are difficult to decipher without a comprehensive systems biology approach. Nevertheless, machine learning and bioinformatics have identified gene signatures associated with macrophages and mitochondrial biomarkers, both of which can be used to differentiate and individualize patients with sepsis-ARDS (167). However, translating these findings into clinically effective metabolic or cell-based therapies demands rigorous validation and optimization.

Another critical challenge is the temporal dynamics of metabolic reprogramming during sepsis progression. Early metabolic shifts may differ substantially from those in later immunosuppressive phases, necessitating time-sensitive interventions. Impaired alveolar macrophage HSD-1 activity compromises local glucocorticoid activation, exacerbating inflammation and mortality in sepsis-related ARDS, indicating that restoring metabolic enzyme function could enhance endogenous anti-inflammatory responses (75). In addition, retinoic acid deficiency can be considered a syndrome in which autacoids and endogenous metabolic mediators become maladaptive, suggesting that modulation of retinoic acid synthesis could prevent lethal immune dysregulation and cytokine storms in sepsis and ARDS (168).

Future research studies should integrate multi-omics data analysis, complex computational modeling, and confirmatory experimentation to accurately map the metabolic-immune axis. This will enable the production of personalized metabolic interventions, with the potential to deliver metabolic modulators or engineered cytotherapy tuned to metabolism. Additionally, combination therapies that target both metabolic pathways and immune checkpoints could be additive or synergistic.

## 3 Conclusion

To summarize, the complex metabolic pattern of macrophages in sepsis-associated ARDS is a critical and dynamic course that powerfully modulates and regulates disease development and immune response. From a clinical specialist’s viewpoint, the associations of macrophages, which pass through a “metabolic hurricane” in the early phases and then progress to the second “immune evolution,” illustrate the distinct acclimatizations of macrophage activation and metabolic re-routing. Eventually, this two-phase adaptation not only initiates the inflammatory stage but also determines the balance between an effective immune response and immune system suppression.

The early activation of glycolysis in macrophages is a critical driver of the acute inflammatory response, facilitating rapid energy production and biosynthesis necessary for pathogen clearance. Nevertheless, unchecked high metabolic activity further harms the tissue and exacerbates lung edema. The opposite, the second phase with delayed metabolic activity, underestimates the body’s immune system. In this case, macrophage aggressiveness is significantly reduced, and they are less likely to interfere with reinfections. In most cases, it leads to even serious consequences through the development of secondary insults. Therefore, the metabolic response of cells can be considered a two-component mechanism, implying the opposite regulation of the cell’s metabolic activity.

Emerging evidence on the role of BMMSCs cell-derived exosomes offers promising avenues for therapeutic innovation. By directing macrophage metabolism, exosomes can help maintain immune homeostasis, restrain overactive inflammation, and promote the resolution of lung injury in sepsis-induced ARDS. This development exemplifies a shift towards metabolically focused immunomodulatory interventions rather than traditional, broad-based anti-inflammatory medications.

In a word, future perspectives, the integration of macrophage metabolic profiling into diagnostic frameworks, could enable early identification of patients at risk for immune paralysis or hyperinflammation, allowing for personalized treatment strategies. In addition, the development of pharmacological agents or biologics that selectively modulate macrophage metabolic states offers a more immediate and attainable solution to improve clinical outcomes. However, translating these insights into effective therapies will require rigorous mechanistic studies and well-designed clinical trials to validate safety, efficacy, and the optimal timing of interventions. Ultimately, balancing various research perspectives, from molecular metabolism to immunology and regenerative medicine, will be instrumental in advancing our understanding and management of sepsis-associated ARDS. The interplay between metabolic pathways and immune function in macrophages is complex and highly context-dependent, necessitating a multidisciplinary approach to dissect. Future research focused on understanding the precise signaling networks and metabolic checkpoints that govern macrophage polarization and function within the septic lung environment may provide the necessary insights to develop therapeutic targets.

In conclusion, macrophage metabolic reprogramming plays a vital role in shaping the inflammatory or immunological microenvironment of ARDS. The ability to leverage this breakthrough and develop metabolism-centered clinical or molecular therapy strategies is a forward-looking approach with huge potential to revolutionize patient care. Future studies must bridge the existing discrepancy and incorporate new technologies in order to fully translate the health implications of macrophage metabolic therapy in this crippling disorder.

## References

[1] Wang Z Wang Z . The role of macrophages polarization in sepsis-induced acute lung injury. Front Immunol. (2023) 14:1209438. doi: 10.3389/fimmu.2023.1209438, PMID: 37691951 PMC10483837

[2] Liu SY Ruan H Li SS . HIF-1α: a bridge connecting sepsis and acute respiratory distress syndrome. Eur J Med Res. (2025) 30:827. doi: 10.1186/s40001-025-03107-z, PMID: 40887582 PMC12399009

[3] Wang QL Yang L Liu ZL Peng Y Gao M Deng LT . Sirtuin 6 regulates macrophage polarization to alleviate sepsis-induced acute respiratory distress syndrome via dual mechanisms dependent on and independent of autophagy. Cytotherapy. (2022) 24:149–60. doi: 10.1016/j.jcyt.2021.09.001, PMID: 34920961

[4] Qiu YJ Zhan F Cheng HP Shao M Li XH Bao XW . Targeting Glutamate transport: A breakthrough in mitigating sepsis lung injury. Free Radic Biol Med. (2025) 235:190–9. doi: 10.1016/j.freeradbiomed.2025.04.043, PMID: 40294854

[5] Li Q Zheng H Chen B . Identification of macrophage-related genes in sepsis-induced ARDS using bioinformatics and machine learning. Sci Rep. (2023) 13:9876. doi: 10.1038/s41598-023-37162-5, PMID: 37336980 PMC10279743

[6] Zhang S Liu Y Zhang XL Sun Y Lu ZH . ANKRD22 aggravates sepsis-induced ARDS and promotes pulmonary M1 macrophage polarization. J Transl Autoimmun. (2024) 8:100228. doi: 10.1016/j.jtauto.2023.100228, PMID: 38225946 PMC10788270

[7] Zhu Z Liu L . Exploring the potential role of the cholinergic anti-inflammatory pathway from the perspective of sepsis pathophysiology. J Intensive Care Med. (2025) 40:571–80. doi: 10.1177/08850666251334342, PMID: 40223326

[8] Wang H Xu P Yin K Wang S . The role of m6A modification during macrophage metabolic reprogramming in human diseases and animal models. Front Immunol. (2025) 16:1521196. doi: 10.3389/fimmu.2025.1521196, PMID: 40066451 PMC11891544

[9] Pomeyie K Abrokwah F Boison D Amoani B Kyei F Adinortey CA . Macrophage immunometabolism dysregulation and inflammatory disorders. BioMed Pharmacother. (2025) 188:118142. doi: 10.1016/j.biopha.2025.118142, PMID: 40378771

[10] Xu HP Niu H Wang H Lin J Yao JJ . Knockdown of RTEL1 alleviates chronic obstructive pulmonary disease by modulating M1, M2 macrophage polarization and inflammation. COPD. (2024) 21:2316607. doi: 10.1080/15412555.2024.2316607, PMID: 38420994

[11] Sun M Zeng Z Xu G An S Deng Z Cheng R . Promoting mitochondrial dynamic equilibrium attenuates sepsis-induced acute lung injury by inhibiting proinflammatory polarization of alveolar macrophages. Shock. (2023) 60:603–12. doi: 10.1097/SHK.0000000000002206, PMID: 37647034

[12] Deng H Wu L Liu M Zhu L Chen Y Zhou H . Bone marrow mesenchymal stem cell-derived exosomes attenuate LPS-induced ARDS by modulating macrophage polarization through inhibiting glycolysis in macrophages. Shock. (2020) 54:828–43. doi: 10.1097/SHK.0000000000001549, PMID: 32433208

[13] Sonobe S Kitabatake M Hara A Konda M Ouji-Sageshima N Terada-Ikeda C . The critical role of the histone modification enzyme setdb2 in the pathogenesis of acute respiratory distress syndrome. Shock. (2023) 60:137–45. doi: 10.1097/SHK.0000000000002145, PMID: 37195726 PMC10417228

[14] Zhao D Wang C Liu X Liu N Zhuang S Zhang Q . CircN4bp1 Facilitates Sepsis-Induced Acute Respiratory Distress Syndrome through Mediating Macrophage Polarization via the miR-138-5p/EZH2 Axis. Mediators Inflamm. (2021) 2021:7858746. doi: 10.1155/2021/7858746, PMID: 35002536 PMC8739551

[15] Yan S Zhou M Zheng X Xing Y Dong J Yan M . Anti-inflammatory effect of curcumin on the mouse model of myocardial infarction through regulating macrophage polarization. Mediators Inflamm. (2021) 2021:9976912. doi: 10.1155/2021/9976912, PMID: 34462629 PMC8403049

[16] Ahmad F . Medicinal nicotine in COVID-19 acute respiratory distress syndrome, the new corticosteroid. World J Crit Care Med. (2022) 11:228–35. doi: 10.5492/wjccm.v11.i4.228, PMID: 36051943 PMC9305679

[17] Alqassim EY Sharma S Khan H Emmons TR Gomez EC Alahmari A . RNA editing enzyme APOBEC3A promotes pro-inflammatory M1 macrophage polarization. Commun Biol. (2021) 4:102. doi: 10.1038/s42003-020-01620-x, PMID: 33483601 PMC7822933

[18] Fresta CG Fidilio A Lazzarino G Musso N Grasso M Merlo S . Modulation of pro-oxidant and pro-inflammatory activities of M1 macrophages by the natural dipeptide carnosine. Int J Mol Sci. (2020) 21(3):776. doi: 10.3390/ijms21030776, PMID: 31991717 PMC7038063

[19] Jian L Li C Wang X Sun L Ma Z Zhao J . IL-21 impairs pro-inflammatory activity of M1-like macrophages exerting anti-inflammatory effects on rheumatoid arthritis. Autoimmunity. (2022) 55:75–85. doi: 10.1080/08916934.2021.2007374, PMID: 34842006

[20] Yang J Ni G Xie X Xu Z . RBM15-mediated VEGFA m6A methylation drives M1 pro-inflammatory macrophage polarization and suppresses M2 anti-inflammatory polarization in acute lung injury. Shock. (2025). in press. doi: 10.1097/SHK.0000000000002697, PMID: 40961397

[21] Yurkanova MD Kosheleva NV Teplova AA Timashev PS Vlasova II . Pro-inflammatory properties of M1 phenotypes of human macrophages: prolongation of myeloperoxidase-mediated oxidative stress. Free Radic Res. (2025) 59:452–61. doi: 10.1080/10715762.2025.2519528, PMID: 40525586

[22] Kang YJ . MicroRNA-22 regulates the pro-inflammatory responses and M1 polarization of macrophages by targeting GLUT1 and 4-1BBL. J Immunol Res. (2023) 2023(0):2457006. doi: 10.1155/2023/2457006, PMID: 37469388 PMC10352528

[23] Delprat V Tellier C Demazy C Raes M Feron O Michiels C . Cycling hypoxia promotes a pro-inflammatory phenotype in macrophages via JNK/p65 signaling pathway. Sci Rep. (2020) 10:882. doi: 10.1038/s41598-020-57677-5, PMID: 31964911 PMC6972721

[24] Ye Q Yan T Shen J Shi X Luo F Ren Y . Sulforaphene targets NLRP3 inflammasome to suppress M1 polarization of macrophages and inflammatory response in rheumatoid arthritis. J Biochem Mol Toxicol. (2023) 37:e23362. doi: 10.1002/jbt.23362, PMID: 36988325

[25] Zhao L Tang S Chen F Ren X Han X Zhou X . Regulation of macrophage polarization by targeted metabolic reprogramming for the treatment of lupus nephritis. Mol Med. (2024) 30:96. doi: 10.1186/s10020-024-00866-z, PMID: 38914953 PMC11197188

[26] Song N Pan K Chen L Jin K . Platelet derived vesicles enhance the TGF-beta signaling pathway of M1 macrophage. Front Endocrinol (Lausanne). (2020) 13:868893. doi: 10.3389/fendo.2022.868893, PMID: 35370988 PMC8972998

[27] Pan X Yuan S Xun X Fan Z Xue X Zhang C . Long-term recruitment of endogenous M2 macrophages by platelet lysate-rich plasma macroporous hydrogel scaffold for articular cartilage defect repair. Adv Healthc Mater. (2022) 11:e2101661. doi: 10.1002/adhm.202101661, PMID: 34969180

[28] Wang Y Chai YQ Cai J Huang SS Wang YF Yuan SS . Human adipose tissue lysate-based hydrogel for lasting immunomodulation to effectively improve spinal cord injury repair. Small. (2024) 20:e2304318. doi: 10.1002/smll.202304318, PMID: 38018305

[29] Wang D Bi X Zhao L Xiang S Xi W Yang S . Targeting SphK1/S1PR3 axis ameliorates sepsis-induced multiple organ injury via orchestration of macrophage polarization and glycolysis. Biochim Biophys Acta Mol Cell Res. (2025) 1872:119877. doi: 10.1016/j.bbamcr.2024.119877, PMID: 39549732

[30] Chen X Liu Y Gao Y Shou S Chai Y . The roles of macrophage polarization in the host immune response to sepsis. Int Immunopharmacol. (2021) 96:107791. doi: 10.1016/j.intimp.2021.107791, PMID: 34162154

[31] Chen L Yang J Zhang M Fu D Luo H Yang X . SPP1 exacerbates ARDS via elevating Th17/Treg and M1/M2 ratios through suppression of ubiquitination-dependent HIF-1α degradation. Cytokine. (2022) 164:156107. doi: 10.1016/j.cyto.2022.156107, PMID: 36773529

[32] Xiong M Luo R Zhang Z Liu P Peng Q Xu F . IL-27 regulates macrophage ferroptosis by inhibiting the Nrf2/HO1 signaling pathway in sepsis-induced ARDS. Inflammation Res. (2025) 74:39. doi: 10.1007/s00011-024-01986-2, PMID: 39945893

[33] Zhang W Wang Y Li C Xu Y Wang X Wu D . Extracellular CIRP-impaired Rab26 restrains EPOR-mediated macrophage polarization in acute lung injury. Front Immunol. (2021) 12:768435. doi: 10.3389/fimmu.2021.768435, PMID: 34925338 PMC8671298

[34] Liu C Xiao K Xie L . Advances in the regulation of macrophage polarization by mesenchymal stem cells and implications for ALI/ARDS treatment. Front Immunol. (2022) 13:928134. doi: 10.3389/fimmu.2022.928134, PMID: 35880175 PMC9307903

[35] Li X Xiao C Yuan J Chen X Li Q Shen F . Rhein-attenuates LPS-induced acute lung injury via targeting NFATc1/Trem2 axis. Inflammation Res. (2023) 72:1237–55. doi: 10.1007/s00011-023-01746-8, PMID: 37212865 PMC10201049

[36] Xie K Chai YS Lin SH Xu F Wang CJ . Luteolin regulates the differentiation of regulatory T cells and activates IL-10-dependent macrophage polarization against acute lung injury. J Immunol Res. (2021) 2021(0):8883962. doi: 10.1155/2021/8883962, PMID: 33532509 PMC7834791

[37] Tang N Yang Y Xie Y Yang G Wang Q Li C . CD274 (PD-L1) negatively regulates M1 macrophage polarization in ALI/ARDS. Front Immunol. (2024) 15:1344805. doi: 10.3389/fimmu.2024.1344805, PMID: 38440722 PMC10909908

[38] Wang M Wu D Liao X Hu H Gao J Meng L . CPT1A-IL-10-mediated macrophage metabolic and phenotypic alterations ameliorate acute lung injury. Clin Transl Med. (2024) 14:e1785. doi: 10.1002/ctm2.1785, PMID: 39090662 PMC11294017

[39] Wang L Wang D Zhang T Ma Y Tong X Fan H . The role of immunometabolism in macrophage polarization and its impact on acute lung injury/acute respiratory distress syndrome. Front Immunol. (2023) 14:1117548. doi: 10.3389/fimmu.2023.1117548, PMID: 37020557 PMC10067752

[40] Cen H Sun M Zheng B Peng W Wen Q Lin Z . Hyaluronic acid modified nanocarriers for aerosolized delivery of verteporfin in the treatment of acute lung injury. Int J Biol Macromol. (2024) 267:131386. doi: 10.1016/j.ijbiomac.2024.131386, PMID: 38582458

[41] Li X Wei Y Li S Liang J Liu Z Cui Y . Zanubrutinib ameliorates lipopolysaccharide-induced acute lung injury via regulating macrophage polarization. Int Immunopharmacol. (2022) 111:109138. doi: 10.1016/j.intimp.2022.109138, PMID: 35973369

[42] Zhai GY Qie SY Guo QY Qi Y Zhou YJ . sDR5-Fc inhibits macrophage M1 polarization by blocking the glycolysis. J Geriatr Cardiol. (2021) 18:271–80. doi: 10.11909/j.issn.1671-5411.2021.04.003, PMID: 33995506 PMC8100429

[43] Chen R Wang J Dai X Wu S Huang Q Jiang L . Augmented PFKFB3-mediated glycolysis by interferon-γ promotes inflammatory M1 polarization through the JAK2/STAT1 pathway in local vascular inflammation in Takayasu arteritis. Arthritis Res Ther. (2022) 24:266. doi: 10.1186/s13075-022-02960-1, PMID: 36510278 PMC9743547

[44] Zhang Y Zhang C Feng R Meng T Peng W Song J . CXCR4 regulates macrophage M1 polarization by altering glycolysis to promote prostate fibrosis. Cell Commun Signal. (2024) 22:456. doi: 10.1186/s12964-024-01828-y, PMID: 39327570 PMC11426013

[45] Qin F Tan H Yang Y Xu L Yang X . Upregulation of Cullin1 neddylation promotes glycolysis and M1 polarization of macrophage via NF-κB p65 pathway in sepsis. Funct Integr Genomics. (2024) 24:204. doi: 10.1007/s10142-024-01483-z, PMID: 39476129

[46] Zhang J Yuan Z Li X Wang F Wei X Kang Y . Activation of the JNK/COX-2/HIF-1α axis promotes M1 macrophage via glycolytic shift in HIV-1 infection. Life Sci Alliance. (2023) 6(12):e202302148. doi: 10.26508/lsa.202302148, PMID: 37798121 PMC10556724

[47] Wu KKL Xu X Wu M Li X Hoque M Hoi G . MDM2 induces pro-inflammatory and glycolytic responses in M1 macrophages by integrating iNOS-nitric oxide and HIF-1α pathways in mice. Nat Commun. (2024) 15:8624. doi: 10.1038/s41467-024-53006-w, PMID: 39366973 PMC11452520

[48] Li Y Zhu L Liu L Li B . Role of macrophage PKM2 in inflammation and tumor progression and its targeted therapy. Biochim Biophys Acta Rev Cancer. (2025) 1880:189478. doi: 10.1016/j.bbcan.2025.189478, PMID: 41101510

[49] Huangfu N Zheng W Xu Z Wang S Wang Y Cheng J . RBM4 regulates M1 macrophages polarization through targeting STAT1-mediated glycolysis. Int Immunopharmacol. (2020) 83:106432. doi: 10.1016/j.intimp.2020.106432, PMID: 32248017

[50] Cheng J Yu Y Zong S Cai W Wang Y Song Y . Berberine ameliorates collagen-induced arthritis in mice by restoring macrophage polarization via AMPK/mTORC1 pathway switching glycolytic reprogramming. Int Immunopharmacol. (2023) 124:111024. doi: 10.1016/j.intimp.2023.111024, PMID: 37827054

[51] Li J Chen X Song S Jiang W Geng T Wang T . Hexokinase 2-mediated metabolic stress and inflammation burden of liver macrophages via histone lactylation in MASLD. Cell Rep. (2025) 44:115350. doi: 10.1016/j.celrep.2025.115350, PMID: 40014451

[52] Hou Y Wei D Zhang Z Guo H Li S Zhang J . FABP5 controls macrophage alternative activation and allergic asthma by selectively programming long-chain unsaturated fatty acid metabolism. Cell Rep. (2022) 41:111668. doi: 10.1016/j.celrep.2022.111668, PMID: 36384126

[53] An L Lu M Xu W Chen H Feng L Xie T . Qingfei oral liquid alleviates RSV-induced lung inflammation by promoting fatty-acid-dependent M1/M2 macrophage polarization via the Akt signaling pathway. J Ethnopharmacol. (2022) 298:115637. doi: 10.1016/j.jep.2022.115637, PMID: 35970312

[54] Zhang J Li N Hu X . Metabolic reprograming of macrophages: A new direction in traditional Chinese medicine for treating liver failure. J Immunol Res. (2024) 2024(0):5891381. doi: 10.1155/jimr/5891381, PMID: 39741958 PMC11688140

[55] Jiang M Li X Zhang J Lu Y Shi Y Zhu C . Dual inhibition of endoplasmic reticulum stress and oxidation stress manipulates the polarization of macrophages under hypoxia to sensitize immunotherapy. ACS Nano. (2021) 15:14522–34. doi: 10.1021/acsnano.1c04068, PMID: 34414762

[56] Weng SW Wu JC Shen FC Chang YH Su YJ Lian WS . MChaperonin counteracts diet-induced non-alcoholic fatty liver disease by aiding sirtuin 3 in the control of fatty acid oxidation. Diabetologia. (2023) 66:913–30. doi: 10.1007/s00125-023-05869-9, PMID: 36692509

[57] Kumar S Mittal S Gupta P Singh M Chaluvally-Raghavan P Pradeep S . Metabolic reprogramming in tumor-associated macrophages in the ovarian tumor microenvironment. Cancers (Basel). (2022) 14(21):5224. doi: 10.3390/cancers14215224, PMID: 36358644 PMC9656653

[58] Peng N Wang Y Jia P Li J Song C Lin J . Intracellular glutathione cleaved long-targeting chains of cancer cells and macrophages to exposedly activate mitochondria delivery of liposomes for enhancing antitumor efficacy by metabolic reprogramming. ACS Appl Mater Interfaces. (2025) 17:49798–815. doi: 10.1021/acsami.5c09138, PMID: 40833833

[59] Feng T Zhao X Gu P Yang W Wang C Guo Q . Adipocyte-derived lactate is a signalling metabolite that potentiates adipose macrophage inflammation via targeting PHD2. Nat Commun. (2022) 13:5208. doi: 10.1038/s41467-022-32871-3, PMID: 36064857 PMC9445001

[60] Ayyangar U Karkhanis A Tay H Afandi AFB Bhattacharjee O KS L . Metabolic rewiring of macrophages by epidermal-derived lactate promotes sterile inflammation in the murine skin. EMBO J. (2024) 43:1113–34. doi: 10.1038/s44318-024-00039-y, PMID: 38418556 PMC10987662

[61] Pålsson-McDermott EM O’Neill LAJ . Gang of 3: How the Krebs cycle-linked metabolites itaconate, succinate, and fumarate regulate macrophages and inflammation. Cell Metab. (2025) 37:1049–59. doi: 10.1016/j.cmet.2025.03.004, PMID: 40169002

[62] Peace CG O’Neill LA . The role of itaconate in host defense and inflammation. J Clin Invest. (2022) 132(2):e148548. doi: 10.1172/JCI148548, PMID: 35040439 PMC8759771

[63] Shi J Cai C . Research progress on the mechanism of itaconate regulating macrophage immunometabolism. Front Immunol. (2022) 13:937247. doi: 10.3389/fimmu.2022.937247, PMID: 35812373 PMC9259868

[64] Trauelsen M Hiron TK Lin D Petersen JE Breton B Husted AS . Extracellular succinate hyperpolarizes M2 macrophages through SUCNR1/GPR91-mediated Gq signaling. Cell Rep. (2021) 35:109246. doi: 10.1016/j.celrep.2021.109246, PMID: 34133934

[65] Harber KJ de Goede KE Verberk SGS Meinster E de Vries HE van Weeghel M . Succinate is an inflammation-induced immunoregulatory metabolite in macrophages. Metabolites. (2020) 10(9):372. doi: 10.3390/metabo10090372, PMID: 32942769 PMC7569821

[66] Xu M Wang X Li Y Geng X Jia X Zhang L . Arachidonic acid metabolism controls macrophage alternative activation through regulating oxidative phosphorylation in PPARγ Dependent manner. Front Immunol. (2021) 12:618501. doi: 10.3389/fimmu.2021.618501, PMID: 34149684 PMC8211451

[67] Chen J Tang Y Qin D Yu X Tong H Tang C . ALOX5 acts as a key role in regulating the immune microenvironment in intrahepatic cholangiocarcinoma, recruiting tumor-associated macrophages through PI3K pathway. J Transl Med. (2023) 21:923. doi: 10.1186/s12967-023-04804-1, PMID: 38124204 PMC10734103

[68] Li X Li S Zang Z He Y . Yaobishu regulates inflammatory, metabolic, autophagic, and apoptosis pathways to attenuate lumbar disc herniation. Oxid Med Cell Longev. (2022) 2022:3861380. doi: 10.1155/2022/3861380, PMID: 35615578 PMC9125431

[69] Shah SAR Khan MI Jawaid H Qureshi U Ul-Haq Z Hafizur MR . Nicotinamide-cinnamic acid cocktail exerts pancreatic β-cells survival coupled with insulin secretion through ERK1/2 signaling pathway in an animal model of apoptosis. Daru. (2021) 29:483–92. doi: 10.1007/s40199-021-00412-w, PMID: 34495496 PMC8602614

[70] Seim GL Britt EC John SV Yeo FJ Johnson AR Eisenstein RS . Two-stage metabolic remodelling in macrophages in response to lipopolysaccharide and interferon-γ stimulation. Nat Metab. (2019) 1:731–42. doi: 10.1038/s42255-019-0083-2, PMID: 32259027 PMC7108803

[71] An L Zhai Q Tao K Xiong Y Ou W Yu Z . Quercetin induces itaconic acid-mediated M1/M2 alveolar macrophages polarization in respiratory syncytial virus infection. Phytomedicine. (2024) .130:155761. doi: 10.1016/j.phymed.2024.155761, PMID: 38797031

[72] Zhuang J Hai Y Lu X Sun B Fan R Zhang B . A self-assembled metabolic regulator reprograms macrophages to combat cytokine storm and boost sepsis immunotherapy. Res (Wash D C). (2025) 8:663. doi: 10.34133/research.0663, PMID: 40171016 PMC11959697

[73] Borek-Dorosz A Pieczara A Orleanska J Brzozowski K Tipping W Graham D . Raman microscopy reveals how cell inflammation activates glucose and lipid metabolism. Biochim Biophys Acta Mol Cell Res. (2024) 1871:119575. doi: 10.1016/j.bbamcr.2023.119575, PMID: 37689141

[74] Hou C Zhang XR Wei J Wang JN Gao J Wang ZJ . METTL1-mediated m7G methylation of Sarm1 mRNA promotes macrophage inflammatory responses and multiple organ injury. Sci Immunol. (2025) 10:eadv4810. doi: 10.1126/sciimmunol.adv4810, PMID: 40911698

[75] Mahida RY Lax S Bassford C Scott A Parekh D Hardy R . Impaired alveolar macrophage 11β-hydroxysteroid dehydrogenase type 1 reductase activity contributes to increased pulmonary inflammation and mortality in sepsis-related ARDS. Front Immunol. (2023) 14:1159831. doi: 10.3389/fimmu.2023.1159831, PMID: 37180160 PMC10172463

[76] Zhao Q Bi Y Guo J Liu Y Zhong J Pan L . Pristimerin protects against inflammation and metabolic disorder in mice through inhibition of NLRP3 inflammasome activation. Acta Pharmacol Sin. (2021) 42:975–86. doi: 10.1038/s41401-020-00527-x, PMID: 32989235 PMC8149413

[77] Jin K Ma Y Manrique-Caballero CL Li H Emlet DR Li S . Activation of AMP-activated protein kinase during sepsis/inflammation improves survival by preserving cellular metabolic fitness. FASEB J. (2020) 34:7036–57. doi: 10.1096/fj.201901900R, PMID: 32246808 PMC11956121

[78] Lahariya R Anand G Kumari B . Trained Immunity in sepsis: Exploring the molecular link to long-term cardiometabolic disorders. Immunol Res. (2025) 73:139. doi: 10.1007/s12026-025-09698-3, PMID: 41032210

[79] Ding W Huang C Chen J Zhang W Wang M Ji X . Exploring the molecular mechanism by which kaempferol attenuates sepsis-related acute respiratory distress syndrome based on network pharmacology and experimental verification. Curr Comput Aided Drug Des. (2025) 21:166–78. doi: 10.2174/0115734099295805240126043059, PMID: 38321908

[80] Gong F Li R Zheng X Chen W Zheng Y Yang Z . OLFM4 regulates lung epithelial cell function in sepsis-associated ARDS/ALI via LDHA-mediated NF-κB signaling. J Inflammation Res. (2021) 14:7035–51. doi: 10.2147/JIR.S335915, PMID: 34955649 PMC8694847

[81] Rump K Adamzik M . Aquaporins in sepsis- an update. Front Immunol. (2024) 15:1495206. doi: 10.3389/fimmu.2024.1495206, PMID: 39544938 PMC11560437

[82] Urtubia A Piñeiro-Hermida S Alfaro-Arnedo E Beni-Ledesma J Canalejo M de Toro M . IGF1R deficiency mitigates acute lung injury by promoting anti-inflammatory transcriptional profiles. Respir Res. (2025) 26:292. doi: 10.1186/s12931-025-03339-x, PMID: 41126254 PMC12542066

[83] Wang H Fan C Chen X Zhou W Guo L Zhao F . Pyruvate kinase M2 nuclear translocation regulate ferroptosis-associated acute lung injury in cytokine storm. Inflammation. (2024) 47:1667–84. doi: 10.1007/s10753-024-02000-x, PMID: 38483700 PMC11549213

[84] Codo AC Davanzo GG Monteiro LB de Souza GF Muraro SP Virgilio-da-Silva JV . Elevated glucose levels favor SARS-CoV-2 infection and monocyte response through a HIF-1α/glycolysis-dependent axis. Cell Metab. (2020) 32:437–446.e5. doi: 10.1016/j.cmet.2020.07.007, PMID: 32697943 PMC7367032

[85] Yang D Liang H Zhu X Li B Li C Hu G . Farnesoid X receptor protects murine lung against IL-6-promoted ferroptosis induced by polyriboinosinic-polyribocytidylic acid. Am J Respir Cell Mol Biol. (2024) 70:364–78. doi: 10.1165/rcmb.2023-0172OC, PMID: 38300138

[86] WuJS XuCY MoSM WuXM DuZB CheL . Palmitoylated COX-2Cys555 reprogrammed mitochondrial metabolism in pyroptotic inflammatory injury in patients with post-acute COVID-19 syndrome. J Adv Res. (2025). in press. doi: 10.1016/j.jare.2025.05.005, PMID: 40349960

[87] Wang X Yan Q Chen L An Y Lu W Zhang R . Metabolic flux analysis elucidates perfluorooctanoic acid-induced mitochondrial metabolic dysregulation in human lung cells. Environ pollut. (2025) 382:126722. doi: 10.1016/j.envpol.2025.126722, PMID: 40562274

[88] Li X Wu J Sun X Wu Q Li Y Li KC . Autophagy reprograms alveolar progenitor cell metabolism in response to lung injury. Stem Cell Rep. (2020) 14:420–32. doi: 10.1016/j.stemcr.2020.01.008, PMID: 32059792 PMC7066233

[89] Kuang X Niu Z Huang Z Cai X Wang L Zhang Y . GDF15 attenuates sepsis-induced acute lung injury by suppressing the HIF-1α/LDHA pathway. Int Immunopharmacol. (2025) 163:115198. doi: 10.1016/j.intimp.2025.115198, PMID: 40669249

[90] Tang L Zhang W Liao Y Wang W Wu Y Zou Z . Decoding sepsis: unraveling key signaling pathways for targeted therapies. Res (Wash D C). (2025) 8:811. doi: 10.34133/research.0811, PMID: 41041277 PMC12484860

[91] Sabbatinelli J Prattichizzo F Olivieri F Procopio AD Rippo MR Giuliani A . Where metabolism meets senescence: focus on endothelial cells. Front Physiol. (2019) 10:1523. doi: 10.3389/fphys.2019.01523, PMID: 31920721 PMC6930181

[92] Scisciola L Olivieri F Ambrosino C Barbieri M Rizzo MR Paolisso G . On the wake of metformin: Do anti-diabetic SGLT2 inhibitors exert anti-aging effects? Ageing Res Rev. (2023) 92:102131. doi: 10.1016/j.arr.2023.102131, PMID: 37984626

[93] Liu Q Li J Sun X Lin J Yu Z Xiao Y . Immunosenescence and cancer: molecular hallmarks, tumor microenvironment remodeling, and age-specific immunotherapy challenges. J Hematol Oncol. (2025) 18:81. doi: 10.1186/s13045-025-01735-w, PMID: 40846970 PMC12374445

[94] Tejero JD Hesterberg RS Drapela S Ilter D Raizada D Lazure F . Methylmalonic acid induces metabolic abnormalities and exhaustion in CD8+ T cells to suppress anti-tumor immunity. Oncogene. (2025) 44:105–14. doi: 10.1038/s41388-024-03191-1, PMID: 39472497 PMC12904991

[95] Yoon KJ Ahn A Park SH Kwak SH Kwak SE Lee W . Exercise reduces metabolic burden while altering the immune system in aged mice. Aging (Albany NY). (2021) 13:1294–313. doi: 10.18632/aging.202312, PMID: 33406502 PMC7834985

[96] Hazeldine J Withnall E Llibre A Duggal NA Lord JM Sardeli AV . Physical activity modifies the metabolic profile of CD4+ and CD8+ T-cell subtypes at rest and upon activation in older adults. Aging Cell. (2025) 2025:e70104. doi: 10.1111/acel.70104, PMID: 40400170 PMC12266771

[97] Malik JA Affan Khan M Lamba T Adeel Zafar M Nanda S Owais M . Immunosuppressive effects of morphine on macrophage polarization and function. Eur J Pharmacol. (2024) 975:176637. doi: 10.1016/j.ejphar.2024.176637, PMID: 38729416

[98] Oyarce C Vizcaino-Castro A Chen S Boerma A Daemen T . Re-polarization of immunosuppressive macrophages to tumor-cytotoxic macrophages by repurposed metabolic drugs. Oncoimmunology. (2021) 10:1898753. doi: 10.1080/2162402X.2021.1898753, PMID: 33796407 PMC7971325

[99] Meng W Li L Hao Y Tang M Cao C He J . NAD+ Metabolism reprogramming mediates irradiation-induced immunosuppressive polarization of macrophages. Int J Radiat Oncol Biol Phys. (2025) 121:176–90. doi: 10.1016/j.ijrobp.2024.07.2327, PMID: 39127084

[100] Herrada AA Olate-Briones A Lazo-Amador R Liu C Hernández-Rojas B Riadi G . Lymph leakage promotes immunosuppression by enhancing anti-inflammatory macrophage polarization. Front Immunol. (2022) 13:841641. doi: 10.3389/fimmu.2022.841641, PMID: 35663931 PMC9160822

[101] Sezginer O Unver N . Dissection of pro-tumoral macrophage subtypes and immunosuppressive cells participating in M2 polarization. Inflammation Res. (2024) 73:1411–23. doi: 10.1007/s00011-024-01907-3, PMID: 38935134 PMC11349836

[102] Chuang HJ Chen YY Chung YD Huang E Huang CY Lung J . The immunosuppressive receptor CD32b regulation of macrophage polarization and its implications in tumor progression. Int J Mol Sci. (2024) 25(17):9737. doi: 10.3390/ijms25179737, PMID: 39273683 PMC11395990

[103] Phi LTH Cheng Y Funakoshi Y Bertucci F Finetti P Van SJ . AXL promotes inflammatory breast cancer progression by regulating immunosuppressive macrophage polarization. Breast Cancer Res. (2025) 27:70. doi: 10.1186/s13058-025-02015-8, PMID: 40329335 PMC12057249

[104] Chandrakesan P Panneerselvam J May R Weygant N Qu D Berry WR . DCLK1-isoform2 alternative splice variant promotes pancreatic tumor immunosuppressive M2-macrophage polarization. Mol Cancer Ther. (2020) 19:1539–49. doi: 10.1158/1535-7163.MCT-19-0776, PMID: 32371580 PMC7883901

[105] Jiang Z Zhang Y Zhang Y Jia Z Zhang Z Yang J . Cancer derived exosomes induce macrophages immunosuppressive polarization to promote bladder cancer progression. Cell Commun Signal. (2021) 19:93. doi: 10.1186/s12964-021-00768-1, PMID: 34521440 PMC8439012

[106] Sun JL Zhang NP Xu RC Zhang GC Liu ZY Abuduwaili W . Tumor cell-imposed iron restriction drives immunosuppressive polarization of tumor-associated macrophages. J Transl Med. (2021) 19:347. doi: 10.1186/s12967-021-03034-7, PMID: 34389031 PMC8361643

[107] Yang F Akhtar MN Zhang D El-Mayta R Shin J Dorsey JF . An immunosuppressive vascular niche drives macrophage polarization and immunotherapy resistance in glioblastoma. Sci Adv. (2024) 10:eadj4678. doi: 10.1126/sciadv.adj4678, PMID: 38416830 PMC10901371

[108] Qi Z Hu R Qi M Zuo J . Carboxypeptidase Q(CPQ) promotes glioma progression by inducing M2 macrophage polarization and immunosuppression. Discov Oncol. (2025) 16:1216. doi: 10.1007/s12672-025-03003-2, PMID: 40591036 PMC12214068

[109] Zhou F Tao J Gou H Liu S Yu D Zhang J . FSTL1 sustains glioma stem cell stemness and promotes immunosuppressive macrophage polarization in glioblastoma. Cancer Lett. (2024) 611:217400. doi: 10.1016/j.canlet.2024.217400, PMID: 39722404

[110] Xu X Xu P Shen G Peng X Liu Z Chen C . Targeting macrophage polarization by inhibiting Pim2 alleviates inflammatory arthritis via metabolic reprogramming. Cell Mol Immunol. (2025) 22:418–36. doi: 10.1038/s41423-025-01268-9, PMID: 40000906 PMC11955556

[111] Wang P Du X Han Z Zhong J Yuan J Jiang L . Nuclear PHGDH regulates macrophage polarization through transcriptional repression of GLUD1 and GLS2 in breast cancer. Cancer Biol Med. (2025) 22(5):502–24. doi: 10.20892/j.issn.2095-3941.2024.0398, PMID: 40434360 PMC12240184

[112] Yuan YS Li HY Lu H Li GC Cao Z Xu C . Reprogramming mitochondrial metabolism to enhance macrophages polarization by ROS-responsive nanoparticles for osteoarthritis. Biomaterials. (2025) 322:123395. doi: 10.1016/j.biomaterials.2025.123395, PMID: 40403559

[113] Wang S Chu X Liu Z Wang C Fan Z Chen Y . Extracellular matrix stiffness facilitates neurite outgrowth by reprogramming the fatty acid oxidation-dependent macrophage polarization. Biochim Biophys Acta Gen Subj. (2025) 1869:130731. doi: 10.1016/j.bbagen.2024.130731, PMID: 39581511

[114] Padovani CM Wilson RM Rodriguez A Spur BW Yin K . Resolvin D2 attenuates LPS-induced macrophage exhaustion. FASEB J. (2024) 38:e23569. doi: 10.1096/fj.202302521R, PMID: 38551610

[115] Sun HJ Zheng GL Wang ZC Liu Y Bao N Xiao PX . Chicoric acid ameliorates sepsis-induced cardiomyopathy via regulating macrophage metabolism reprogramming. Phytomedicine. (2023) 123:155175. doi: 10.1016/j.phymed.2023.155175, PMID: 37951150

[116] Ji T Zhao T Long SZ Wei CZ Cheng DY Chen J . Microvesicle-transferred mitochondria trigger cGAS-STING and reprogram metabolism of macrophages in sepsis. Microbiol Spectr. (2025) 13:e0078125. doi: 10.1128/spectrum.00781-25, PMID: 40905697 PMC12502667

[117] Luo R Li X Wang D . Reprogramming macrophage metabolism and its effect on NLRP3 inflammasome activation in sepsis. Front Mol Biosci. (2022) 9:917818. doi: 10.3389/fmolb.2022.917818, PMID: 35847986 PMC9276983

[118] Zhao Z Ji J Ming L Luo Z Li M Chen Y . Direct peritoneal resuscitation and melatonin in the treatment of abdominal sepsis-induced lung injury via macrophage metabolic reprogramming. J Pineal Res. (2025) 77:e70066. doi: 10.1111/jpi.70066, PMID: 40662254

[119] Huang W Wang L Huang Z Sun Z Zheng B . Peroxiredoxin 3 has a crucial role in the macrophage polarization by regulating mitochondrial homeostasis. Respir Res. (2024) 25:110. doi: 10.1186/s12931-024-02739-9, PMID: 38431661 PMC10909251

[120] Shao L Wang C Zhang Y . Research progress on metabolic reprogramming of innate immune cells involved in immune-regulation of sepsis. Zhonghua Wei Zhong Bing Ji Jiu Yi Xue. (2019) 31:910–2. doi: 10.3760/cma.j.issn.2095-4352.2019.07.023, PMID: 31441421

[121] Wang D Liu M Lyu X . Research progress in glycogen metabolism reprogramming in sepsis associated immune cells. Zhonghua Wei Zhong Bing Ji Jiu Yi Xue. (2019) 31:1167–9. doi: 10.3760/cma.j.issn.2095-4352.2019.09.023, PMID: 31657347

[122] Yao C Zhu H Ji B Guo H Liu Z Yang N . rTM reprograms macrophages via the HIF-1α/METTL3/PFKM axis to protect mice against sepsis. Cell Mol Life Sci. (2024) 81:456. doi: 10.1007/s00018-024-05489-5, PMID: 39549085 PMC11569104

[123] Fang C Ren P Bian G Wang J Bai J Huang J . Enhancing Spns2/S1P in macrophages alleviates hyperinflammation and prevents immunosuppression in sepsis. EMBO Rep. (2023) 24:e56635. doi: 10.15252/embr.202256635, PMID: 37358015 PMC10398662

[124] Chen F Wang N Liao J Jin M Qu F Wang C . Esculetin rebalances M1/M2 macrophage polarization to treat sepsis-induced acute lung injury through regulating metabolic reprogramming. J Cell Mol Med. (2024) 28:e70178. doi: 10.1111/jcmm.70178, PMID: 39535339 PMC11558263

[125] Li X Long Y Zhu Y Gu J Zhou P Miao C . Endothelial-derived CCL7 promotes macrophage polarization and aggravates septic acute lung injury via CCR1-mediated STAT1 succinylation. Adv Sci (Weinh). (2025) 12:e06209. doi: 10.1002/advs.202506209, PMID: 40755420 PMC12520477

[126] Xu FT Ling Y Wei HX Meng L Yin D Lai ZH . BMSC-EXO deliver JKAP to restore Th17/Treg balance via AKT/ERK, alleviating rheumatoid arthritis. iScience. (2025) 28:112832. doi: 10.1016/j.isci.2025.112832, PMID: 40687841 PMC12271589

[127] He Y Ji D Lu W Li F Huang X Huang R . Bone marrow mesenchymal stem cell-derived exosomes induce the Th17/Treg imbalance in immune thrombocytopenia through miR-146a-5p/IRAK1 axis. Hum Cell. (2021) 34:1360–74. doi: 10.1007/s13577-021-00547-7, PMID: 34052997

[128] Rong Y Lu W Huang X Ji D Tang D Huang R . Exosomal miR-146a-5p derived from bone marrow mesenchymal stromal cells regulate Th1/Th2 balance and alleviates immune thrombocytopenia in pregnancy. Hum Cell. (2024) 38:31. doi: 10.1007/s13577-024-01162-y, PMID: 39699695

[129] He JG Wu XX Li S Yan D Xiao GP Mao FG . Exosomes derived from microRNA-540-3p overexpressing mesenchymal stem cells promote immune tolerance via the CD74/nuclear factor-kappaB pathway in cardiac allograft. World J Stem Cells. (2024) 16:1022–46. doi: 10.4252/wjsc.v16.i12.1022, PMID: 39734479 PMC11669987

[130] Lv Z Duan S Zhou M Gu M Li S Wang Y . Mouse bone marrow mesenchymal stem cells inhibit sepsis-induced lung injury in mice via exosomal SAA1. Mol Pharm. (2022) 19:4254–63. doi: 10.1021/acs.molpharmaceut.2c00542, PMID: 36173129

[131] Huang Y Lu D Ma W Liu J Ning Q Tang F . miR-223 in exosomes from bone marrow mesenchymal stem cells ameliorates rheumatoid arthritis via downregulation of NLRP3 expression in macrophages. Mol Immunol. (2022) 143:68–76. doi: 10.1016/j.molimm.2022.01.002, PMID: 35042119

[132] Yu F Yang J Chen J Wang X Cai Q He Y . Bone marrow mesenchymal stem cell-derived exosomes alleviate peritoneal dialysis-associated peritoneal injury. Stem Cells Dev. (2023) 32:197–211. doi: 10.1089/scd.2022.0244, PMID: 36691747

[133] Bai Z Hu H Hu F Ji J Ji Z . Bone marrow mesenchymal stem cellsderived exosomes stabilize atherosclerosis through inhibiting pyroptosis. BMC Cardiovasc Disord. (2023) 23:441. doi: 10.1186/s12872-023-03453-y, PMID: 37679676 PMC10486039

[134] Yu H Xu Z Qu G Wang H Lin L Li X . Hypoxic preconditioning enhances the efficacy of mesenchymal stem cells-derived conditioned medium in switching microglia toward anti-inflammatory polarization in ischemia/reperfusion. Cell Mol Neurobiol. (2021) 41:505–24. doi: 10.1007/s10571-020-00868-5, PMID: 32424775 PMC11448619

[135] Li W Xu Y Chen W . Bone mesenchymal stem cells deliver exogenous lncRNA CAHM via exosomes to regulate macrophage polarization and ameliorate intervertebral disc degeneration. Exp Cell Res. (2022) 421:113408. doi: 10.1016/j.yexcr.2022.113408, PMID: 36334792

[136] Xu S Cheuk YC Jia Y Chen T Chen J Luo Y . Bone marrow mesenchymal stem cell-derived exosomal miR-21a-5p alleviates renal fibrosis by attenuating glycolysis by targeting PFKM. Cell Death Dis. (2022) 13:876. doi: 10.1038/s41419-022-05305-7, PMID: 36253358 PMC9576726

[137] Wang J Hu Y Wang Z Fan C Liu Y Xie Y . Exosomes derived from human gingival mesenchymal stem cells induce metabolic reprogramming of inflammatory macrophages. J Clin Periodontol. (2025) 52:1196–210. doi: 10.1111/jcpe.14184, PMID: 40388972

[138] Liu X Gao C Wang Y Niu L Jiang S Pan S . BMSC-derived exosomes ameliorate LPS-induced acute lung injury by miR-384-5p-controlled alveolar macrophage autophagy. Oxid Med Cell Longev. (2021) 2021:9973457. doi: 10.1155/2021/9973457, PMID: 34234888 PMC8216833

[139] Deng H Zhu L Zhang Y Zheng L Hu S Zhou W . Differential lung protective capacity of exosomes derived from human adipose tissue, bone marrow, and umbilical cord mesenchymal stem cells in sepsis-induced acute lung injury. Oxid Med Cell Longev. (2022) 2022:7837837. doi: 10.1155/2022/7837837, PMID: 35265265 PMC8898768

[140] Chen F Chen Z Wu HT Chen XX Zhan P Wei ZY . Mesenchymal stem cell-derived exosomes attenuate murine cytomegalovirus-infected pneumonia via NF-κB/NLRP3 signaling pathway. Viruses. (2024) 16. doi: 10.3390/v16040619, PMID: 38675960 PMC11054941

[141] Tang X Xue J Li X Zhang J Zhou J . G6PC1 expression as a prognostic biomarker associated with metabolic reprogramming and tumor microenvironment in hepatocellular carcinoma. Front Immunol. (2025) 16:1623315. doi: 10.3389/fimmu.2025.1623315, PMID: 40821770 PMC12354593

[142] Gao B Zheng D Liu H Guo Y Ye Y Chen Z . Asparagine synthetase modulates glutaminase inhibitor sensitivity through metabolic reprogramming and serves as a prognostic biomarker in hepatocellular carcinoma. Redox Biol. (2025) 86:103813. doi: 10.1016/j.redox.2025.103813, PMID: 40779838 PMC12356003

[143] Li D Liang J Yang W Guo W Song W Zhang W . A distinct lipid metabolism signature of acute myeloid leukemia with prognostic value. Front Oncol. (2022) 12:876981. doi: 10.3389/fonc.2022.876981, PMID: 35957912 PMC9359125

[144] Wei L Ji L Han S Xu M Yang X . Construction and validation of a prognostic model of metabolism-related genes driven by somatic mutation in bladder cancer. Front Biosci (Landmark Ed). (2023) 28:242. doi: 10.31083/j.fbl2810242, PMID: 37919060

[145] Huo J Wu L Zang Y Dong H Liu X He F . Eight-gene metabolic signature related with tumor-associated macrophages predicting overall survival for hepatocellular carcinoma. BMC Cancer. (2021) 21:31. doi: 10.1186/s12885-020-07734-z, PMID: 33413205 PMC7789516

[146] Liu Y Yan Z Liu C Yang R Zheng Q Jian J . Integrated RNA sequencing analysis and machine learning identifies a metabolism-related prognostic signature in clear cell renal cell carcinoma. Sci Rep. (2025) 15:1691. doi: 10.1038/s41598-025-85618-7, PMID: 39799252 PMC11724983

[147] Shen T Wang T . Metabolic reprogramming in COVID-19. Int J Mol Sci. (2021) 22. doi: 10.3390/ijms222111475, PMID: 34768906 PMC8584248

[148] Rudiansyah M Jasim SA Mohammad Pour ZG Athar SS Jeda AS Doewes RI . Coronavirus disease 2019 (COVID-19) update: From metabolic reprogramming to immunometabolism. J Med Virol. (2022) 94:4611–27. doi: 10.1002/jmv.27929, PMID: 35689351 PMC9350347

[149] Liu Y Xu R Gu H Zhang E Qu J Cao W . Metabolic reprogramming in macrophage responses. biomark Res. (2021) 9:1. doi: 10.1186/s40364-020-00251-y, PMID: 33407885 PMC7786975

[150] Wang X Zhang S Xue D Neculai D Zhang J . Metabolic reprogramming of macrophages in cancer therapy. Trends Endocrinol Metab. (2025) 36:660–76. doi: 10.1016/j.tem.2024.08.009, PMID: 39304355

[151] Wang Y Wang D Yang L Zhang Y . Metabolic reprogramming in the immunosuppression of tumor-associated macrophages. Chin Med J (Engl). (2022) 135:2405–16. doi: 10.1097/CM9.0000000000002426, PMID: 36385099 PMC9945195

[152] Fan H Lu D Yin X Sun H Wang Z Sun H . The metabolic regulation of tumor-associated macrophage reprogramming. Mol Immunol. (2025) 187:198–209. doi: 10.1016/j.molimm.2025.10.006, PMID: 41092688

[153] Cheng S Li Y Sun X Liu Z Guo L Wu J . The impact of glucose metabolism on inflammatory processes in sepsis-induced acute lung injury. Front Immunol. (2024) 15:1508985. doi: 10.3389/fimmu.2024.1508985, PMID: 39712019 PMC11659153

[154] Prajumwongs P Titapun A Thanasukarn V Jareanrat A Khuntikeo N Namwat N . Identification of serum metabolite biomarkers and metabolic reprogramming mechanisms to predict recurrence in cholangiocarcinoma. Sci Rep. (2025) 15:12782. doi: 10.1038/s41598-025-97641-9, PMID: 40229491 PMC11997029

[155] Li W Lu Z Pan D Zhang Z He H Wu J . Gene expression analysis reveals prognostic biomarkers of the tyrosine metabolism reprogramming pathway for prostate cancer. J Oncol. (2022) 2022:5504173. doi: 10.1155/2022/5504173, PMID: 35847355 PMC9279037

[156] Afonso J Gonçalves C Costa M Ferreira D Santos L Longatto-Filho A . Glucose metabolism reprogramming in bladder cancer: hexokinase 2 (HK2) as prognostic biomarker and target for bladder cancer therapy. Cancers (Basel). (2023) 15(3):982. doi: 10.3390/cancers15030982, PMID: 36765947 PMC9913750

[157] Wu GW Hsieh YH Chien YC Bai LY Yu YL . Novel insights into PGM2L1 as a prognostic biomarker in cholangiocarcinoma: implications for metabolic reprogramming and tumor microenvironment modulation. Int J Med Sci. (2025) 22:1158–66. doi: 10.7150/ijms.106566, PMID: 40027181 PMC11866533

[158] Weng X Huang Y Fu Z Liu X Xie F Wang J . METTL1-driven nucleotide metabolism reprograms the immune microenvironment in hepatocellular carcinoma: a multi-omics approach for prognostic biomarker discovery. Front Immunol. (2025) 16:1582203. doi: 10.3389/fimmu.2025.1582203, PMID: 40330476 PMC12052905

[159] Wang L Dou X Xie L Wang L Dou X Xie L . Metabolic landscape of osteosarcoma: reprogramming of lactic acid metabolism and metabolic communication. Front Biosci (Landmark Ed). (2024) 29:83. doi: 10.31083/j.fbl2902083, PMID: 38420794

[160] Gao H Ding M Liu Y Wang Y Zhao S Chen J . Reprogramming immunity with itaconate: metabolic mechanisms and therapeutic perspectives. Inflammation Res. (2025) 74:128. doi: 10.1007/s00011-025-02087-4, PMID: 40956430

[161] Chen Y Wu Y Zhu L Chen C Xu S Tang D . METTL3-mediated N6-methyladenosine modification of trim59 mRNA protects against sepsis-induced acute respiratory distress syndrome. Front Immunol. (2022) 13:897487. doi: 10.3389/fimmu.2022.897487, PMID: 35693774 PMC9174697

[162] Liu N Zhong Y Pang X Li M Cannon RD Mei L . The nano-windmill exerts superior anti-inflammatory effects via reducing choline uptake to inhibit macrophage activation. Cell Prolif. (2023) 56:e13470. doi: 10.1111/cpr.13470, PMID: 37051938 PMC10542611

[163] Lee SE Kim IH Kang YC Kim Y Yu SH Yeo JS . Mitochondrial transplantation attenuates lipopolysaccharide-induced acute respiratory distress syndrome. BMC Pulm Med. (2024) 24:477. doi: 10.1186/s12890-024-03304-2, PMID: 39334020 PMC11437886

[164] Gu WJ Zhao FZ Huang W Zhu MG Huang HY Yin HY . Selenium nanoparticles activate selenoproteins to mitigate septic lung injury through miR-20b-mediated RORγt/STAT3/Th17 axis inhibition and enhanced mitochondrial transfer in BMSCs. J Nanobiotechnology. (2025) 23:226. doi: 10.1186/s12951-025-03312-2, PMID: 40114196 PMC11924768

[165] Trovato FM Zia R Artru F Mujib S Jerome E Cavazza A . Lysophosphatidylcholines modulate immunoregulatory checkpoints in peripheral monocytes and are associated with mortality in people with acute liver failure. J Hepatol. (2023) 78:558–73. doi: 10.1016/j.jhep.2022.10.031, PMID: 36370949

[166] Zheng M Zhu Y Wei K Pu H Peng R Xiao J . Metformin attenuates the inflammatory response via the regulation of synovial M1 macrophage in osteoarthritis. Int J Mol Sci. (2023) 24(6):5355. doi: 10.3390/ijms24065355, PMID: 36982442 PMC10049635

[167] Sun W Tu S . Screening of mitochondrial-related biomarkers connected with immune infiltration for acute respiratory distress syndrome through WGCNA and machine learning. Med (Baltimore). (2025) 104:e41497. doi: 10.1097/MD.0000000000041497, PMID: 40068062 PMC11903030

[168] Sarohan AR . COVID-19: endogenous retinoic acid theory and retinoic acid depletion syndrome. Med Hypotheses. (2020) 144:110250. doi: 10.1016/j.mehy.2020.110250, PMID: 33254555 PMC7481114

